# Habitat variability and faunal zonation at the Ægir Ridge, a canyon-like structure in the deep Norwegian Sea

**DOI:** 10.7717/peerj.13394

**Published:** 2022-06-15

**Authors:** Saskia Brix, Stefanie Kaiser, Anne-Nina Lörz, Morgane Le Saout, Mia Schumacher, Frederic Bonk, Hronn Egilsdottir, Steinunn Hilma Olafsdottir, Anne Helene S. Tandberg, James Taylor, Simon Tewes, Joana R. Xavier, Katrin Linse

**Affiliations:** 1 Senckenberg am Meer, German Center for Marine Biodiversity Research (DZMB), Senckenberg Nature Research Society, Hamburg, Germany; 2 Faculty of Biology and Environmental Protection, Department of Invertebrate Zoology and Hydrobiology, Łódź, Poland; 3 INES Integrated Environmental Solutions UG, Wilhelmshaven, Niedersachsen, Germany; 4 Institute for Marine Ecosystems and Fisheries Science, Center for Earth System Research and Sustainability (CEN), University of Hamburg, Hamburg, Germany; 5 GEOMAR Helmholtz Centre for Ocean Research, Kiel, Germany; 6 Marine and Freshwater Research Institute, Hafnarfjordur, Iceland; 7 University Museum, University of Bergen, Bergen, Norway; 8 Bundesamt für Seeschiffahrt und Hydrographie, Hamburg, Germany; 9 CIIMAR–Interdisciplinary Centre of Marine and Environmental Research of the University of Portro, Matosinhos, Portugal; 10 Department of Biological Sciences, University of Bergen, Bergen, Norway; 11 British Antarctic Survey, Cambridge, United Kingdom

**Keywords:** Iceland, Deep sea, Marine invertebrates, Arctic circle, EBSA, VME, ROV transects, Banana hole, Sponge gardens, Soft corals

## Abstract

The Ægir Ridge System (ARS) is an ancient extinct spreading axis in the Nordic seas extending from the upper slope east of Iceland (∼550 m depth), as part of its Exclusive Economic Zone (EEZ), to a depth of ∼3,800 m in the Norwegian basin. Geomorphologically a rift valley, the ARS has a canyon-like structure that may promote increased diversity and faunal density. The main objective of this study was to characterize benthic habitats and related macro- and megabenthic communities along the ARS, and the influence of water mass variables and depth on them. During the IceAGE3 expedition (Icelandic marine Animals: Genetics and Ecology) on RV Sonne in June 2020, benthic communities of the ARS were surveyed by means of a remotely-operated vehicle (ROV) and epibenthic sledge (EBS). For this purpose, two working areas were selected, including abyssal stations in the northeast and bathyal stations in the southwest of the ARS. Video and still images of the seabed were usedtoqualitatively describebenthic habitats based on the presence of habitat-forming taxa and the physical environment. Patterns of diversity and community composition of the soft-sediment macrofauna, retrieved from the EBS, were analyzed in a semiquantitative manner. These biological data were complemented by producing high-resolution bathymetric maps using the vessel’s multi-beam echosounder system. As suspected, we were able to identify differences in species composition and number of macro- and megafaunal communities associated with a depth gradient. A biological canyon effect became evident in dense aggregates of megafaunal filter feeders and elevated macrofaunal densities. Analysis of videos and still images from the ROV transects also led to the discovery of a number ofVulnerable Marine Ecosystems (VMEs) dominated by sponges and soft corals characteristic of the Arctic region. Directions for future research encompass a more detailed, quantitative study of the megafauna and more coherent sampling over the entire depth range in order to fully capture the diversity of the habitats and biota of the region. The presence of sensitive biogenic habitats, alongside seemingly high biodiversity and naturalness are supportive of ongoing considerations of designating part of the ARS as an “Ecologically and Biologically Significant Area” (EBSA).

## Introduction

Life on the deep seafloor beyond the shelf break is undoubtedly rich and contains a significant fraction of the global marine biodiversity (Appeltans et al., 2012; Danovaro, Snelgrove & Tyler, 2014). The deep sea also holds an abundance of geomorphic features, including seamounts, canyons, troughs, ridges, trenches, and abyssal plains that are anticipated to represent distinct types of benthic habitats (Ramirez-Llodra et al., 2010; Harris & Baker, 2011). A combination of factors and processes are considered to have shaped the diversity and distribution of today’s deep-sea fauna. Among these, bathymetry, temperature, salinity, oxygen, hydrostatic pressure, organic matter flux, substrate type, and seabed geomorphology stand out as key environmental descriptors (Levin et al., 2001). The variety of geological features (or geodiversity) creates a heterogeneous environment that promotes high diversity of habitats and species on a range of scales. Bathymetric discontinuities, such as seamounts, submarine canyons, and ridges, can have a significant impact on hydrodynamics by diverting current flow or changing current velocity. For example, unique hydrographic features of canyons can lead to increased flux and channeling of organic matter from surface waters to the sea floor, making them areas of augmented benthic biomass and productivity, but also promoting biodiversity in several faunal taxa (Schlacher et al., 2007; De Leo et al., 2010; Vetter, Smith & De Leo, 2010; Leduc et al., 2020). Furthermore, topographical and biogenic structures, such as boulders or corals, as well as disturbance or ephemeral food patches contribute to increased local-scale heterogeneity and thus biodiversity in the deep sea (McClain & Barry, 2010; Vanreusel et al., 2010; Riehl et al., 2020).

**Figure 1:**
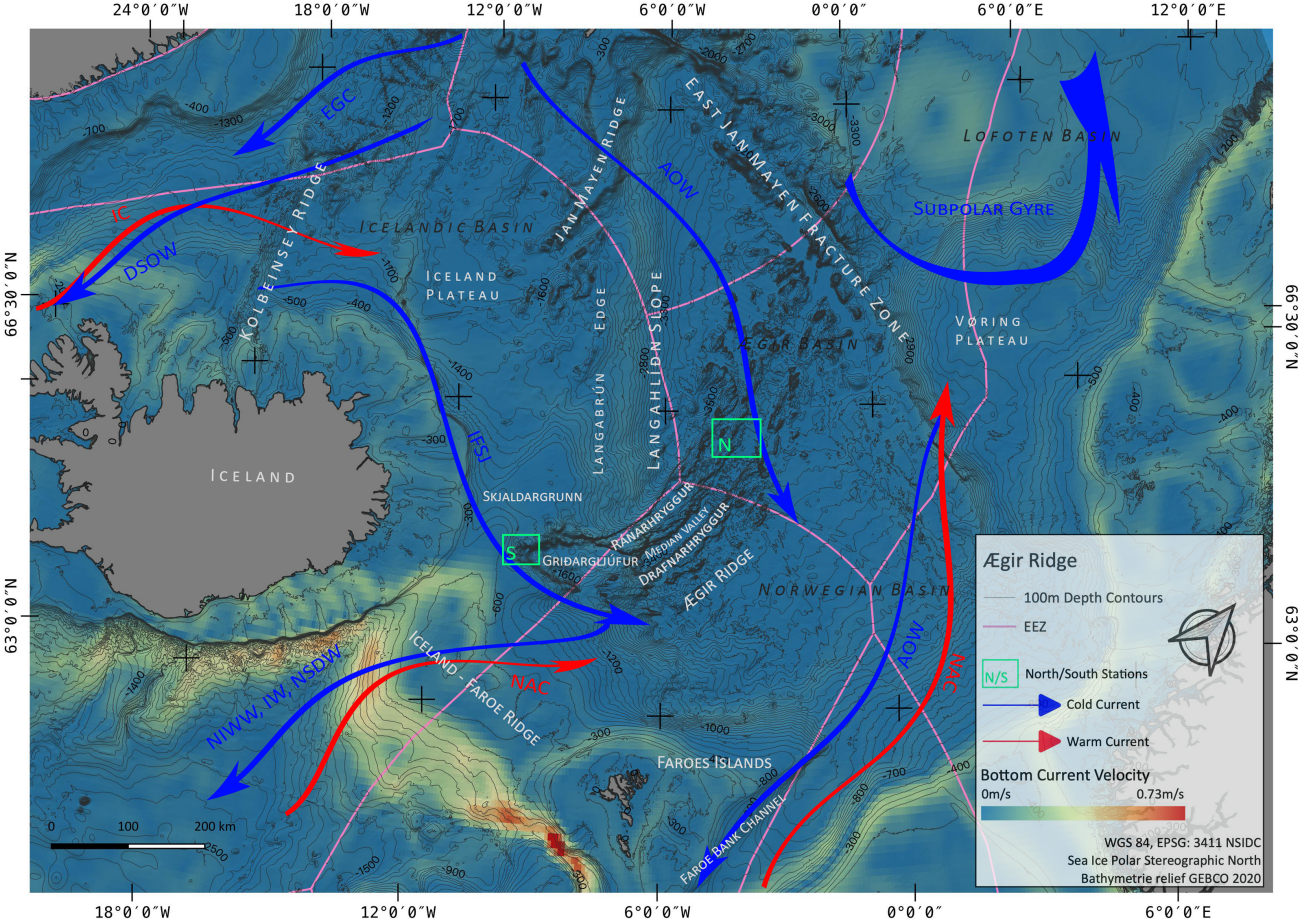
Overview map. Map of the study region. Green boxes ‘N’ and ‘S’ indicated the sampling areas ‘North’ and ‘South’ at the Ægir Ridge. Blue arrows indicate dense, cold water currents, red arrows indicate warmer water currents. AOW–Arctic Overflow Water, DSOW –Denmark Strait Overflow Water, EGC -East Greenland Current, NAC–North Atlantic Current. Map produced with software QGIS 3.16.4.; bathymetry obtained from GEBCO Compilation Group; bottom current velocity obtained from Copernicus locator model (Copernicus Marine Service, 2021).

Understanding species’ spatial distributions and their relationships to the abiotic seafloor environment makes a fundamental contribution to marine spatial planning and serves as a basis for monitoring potential future shifts in biodiversity and biogeographic ranges (Costello, 2009). Typical conservation management ensures sustainable use of marine resources while safeguarding marine life, their habitats, and functions, *i.e.*, preserving the biological and geological heritage of the marine realm (Ware & Downie, 2020). However, there remains a general lack of knowledge about the biological and physical components of deep-sea ecosystems, also due to the fact that a mere fraction of the seabed has been thoroughly mapped to date (Wölfl et al., 2019). To remedy this deficiency, national and international initiatives, including the Seabed 2030 (http://seabed2030.org) project or the Norwegian MAREANO (Marine Areal Database for Norwegian Coasts and Sea Areas) program (Buhl-Mortensen et al., 2015a), are now underway. As part of the German IceAGE3 (SO276) expedition in summer 2020, efforts were made to expand the bathymetry data of the Norwegian basin particularly along the Ægir Ridge system (ARS, Fig. 1) nested therein, and to characterize its associated habitats and macro- and megafaunal communities (Brix et al., 2020). MAREANO is making efforts to collate data in the Norwegian EEZ and beyond, making them publicly available. One focus here is area-wide high-resolution mapping of the Nordic and Barents Seas, with a vast multibeam coverage having already been achieved. The ARS has been mapped at its center and northern rim during the recent bathymetric mappings (2018–2020) of the Norwegian Sea by the Norwegian Mapping authorities (Kartverket, 2007). Using these existing bathymetric data, IceAGE3 intends to fill data gaps and expand the overall high-resolution bathymetric knowledge.

The Nordic seas, including the deep basin of the Norwegian Sea and neighbouring Greenland and Iceland seas, as well as the Barents Sea, with the Greenland-Scotland Ridge (GSR) (including *i.e.*, the Greenland-Iceland Ridge [GIR] and the Iceland-Faroe Ridge [IFR]) as a boundary towards the Atlantic, are known to host various kinds of different water masses. The prevailing current systems are supplied by the upstreaming North Atlantic Current (NAC) and the Irminger Current (IC) that transport warm, saline water from the Atlantic into the Nordic seas (Fig. 1). The branch of IC that reaches to the northern part of Iceland is often referred as NIIC (North Icelandic Irminger Current). These currents become subject to heat loss and mixing with dense Arctic waters, forming the subpolar gyre as well as boundary current systems (Mauritzen, 1996; Chatterjee et al., 2018; Puerta et al., 2020). The Norwegian Basin region is known to be a deep-water formation area and, although most of the water is trapped here, parts of it return to the northern Atlantic as intermediate overflow plumes (Mauritzen, 1996; Swift & Aagaard, 1982) or as deep boundary currents (Våge et al., 2011), thereby playing a major role for the Atlantic Meridional Overturning Circulation (AMOC) (Semper et al., 2020; Puerta et al., 2020).

The two major downstream (southward transport) pathways are through the Denmark Strait, a deep passage in the GIR, and over the IFR (Swift & Aagaard, 1982; Hansen & Østerhus, 2000; Våge et al., 2011; Semper et al., 2020). The Denmark Strait Overflow Current (DSOC) is a well-studied (*e.g.*, Mauritzen, 1996; Våge et al., 2011; Mastropole et al., 2017) water mass bulk transport comprised of the two East Greenland Current (EGC) branches and the deep North Iceland Jet (NIJ), streaming westward along the continental shelf of Iceland. The Iceland-Scotland Overflow is formed by Arctic Intermediate water masses mainly originated in the Iceland Sea, whereas deep water formed in the Norwegian sea leaves the basin through the Faroe Bank Channel (FBC) (Mauritzen, 1996; Semper et al., 2020). At the southern tip of ARS, called Gríđargljúfur Gorge, it is mainly the newly discovered deep Iceland Faroe Slope Jet (IFSJ) that supplies the local deep-water masses with cold and dense water, streaming eastward along the northern slope of the Greenland-Iceland Ridge (Semper et al., 2020). The upstreaming North Atlantic Current contributes to the local surface water masses at the location and over the extent of ARS, transporting warm and saline water into the Norwegian basin, which itself is part of the subpolar gyre (*e.g.*, Chatterjee et al., 2018).

**Figure 2:**
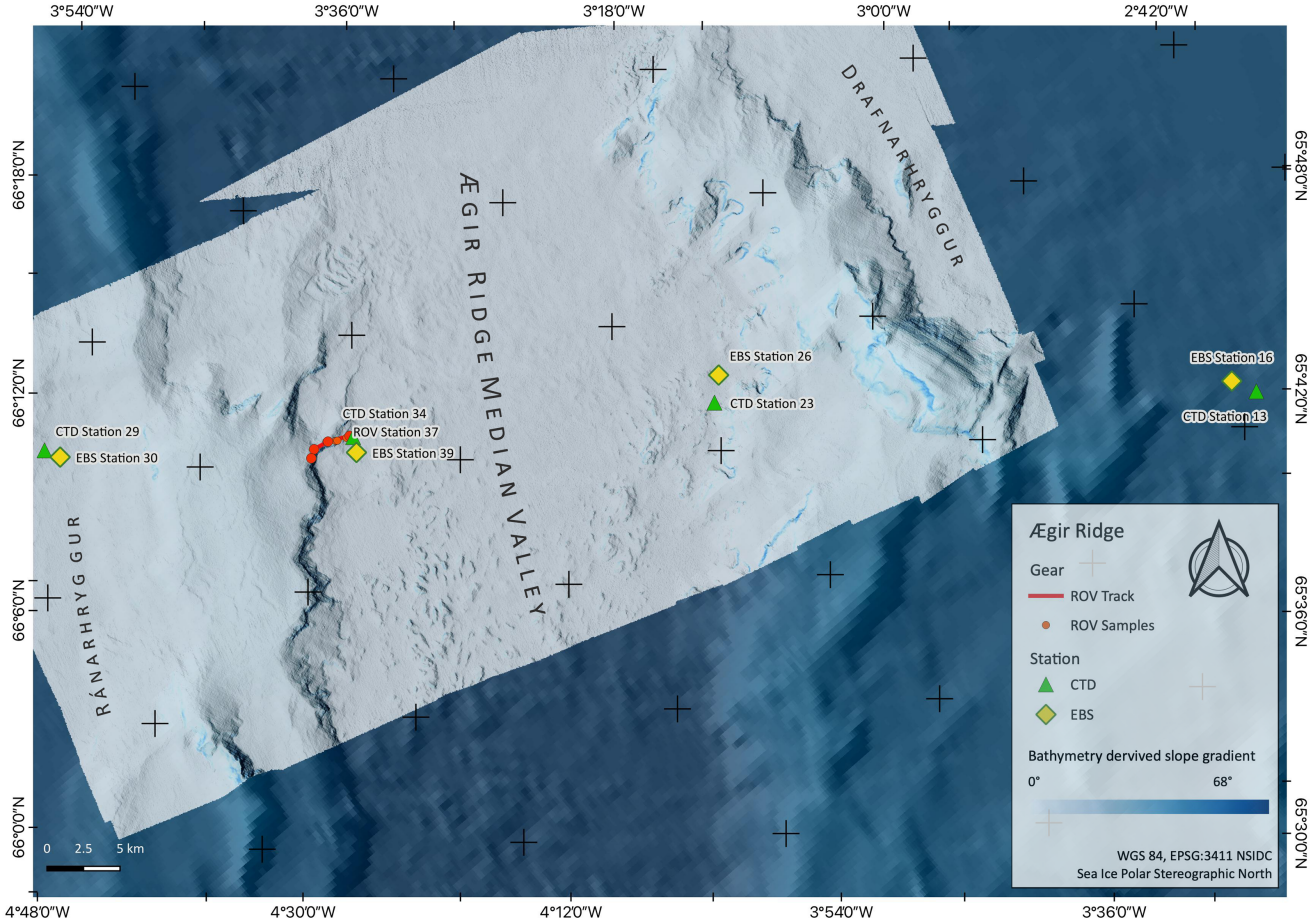
Sampling Area North. Detailed map of sampling area ‘North’ at the Ægir Ridge, including locations of ROV dives, CTD and EBS deployments. Map produced with software QGIS 3.16.4.; bathymetry obtained multibeam survey and processed with QPS Qimera 2.0.

**Figure 3:**
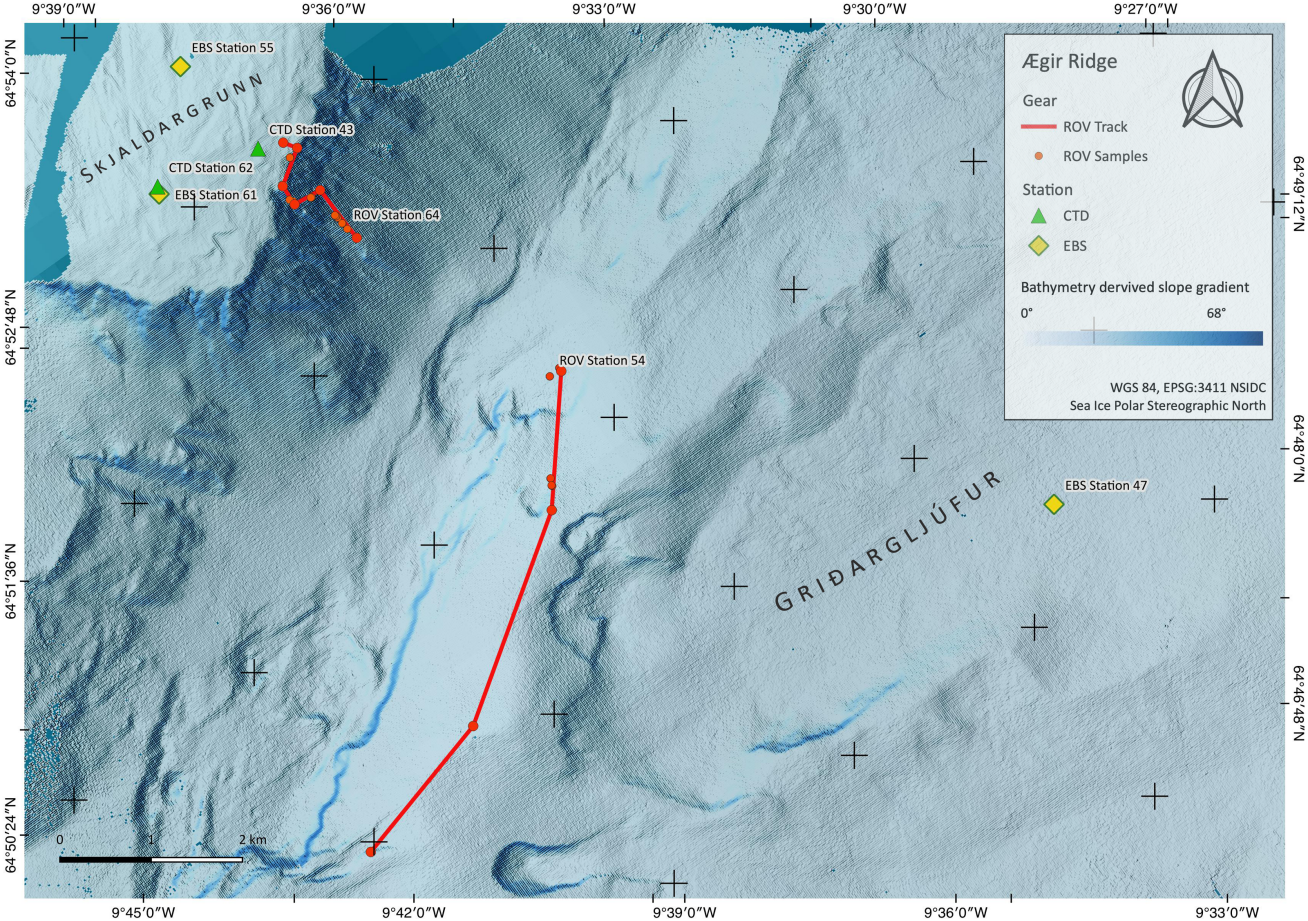
Sampling Area South. Detailed map of the sampling area ‘South’ at the Ægir Ridge, including locations of ROV dives, CTD and EBS deployments. Map produced with software QGIS 3.16.4.; bathymetry obtained multibeam survey and processed with QPS Qimera 2.0.

The ARS (Fig. 1) is an extinct spreading axis in the Nordic seas west of the Norwegian Basin, located south-east of the Jan Mayen microcontinent, bound by the East-Jan-Mayen-Fracture-Zone at its northern flank and the GSR at its southern end on the Iceland Plateau (Blischke et al., 2016). Politically, the northern half of the ARS (Figs. 1 and 2) is located in the high seas in an area beyond national jurisdiction (ABNJ). The southern half of the median valley is within the Faroe exclusive economic zone (EEZ) (Figs. 1 and 3) and the southern extremity belongs to the Iceland EEZ. Together with the Mohns Ridge further north, the ARS represents the first spreading center linked to the continental breakup between Norway and Greenland from 55 Ma to its abortion approximately 25 Ma ago (Talwani & Eldholm, 1977; Gaina, Gernigon & Ball, 2009; MacLeod et al., 2017). Reflection seismic surveys (Uenzelmann-Neben et al., 1992) reveal that the median valley is covered by between 900 and 1,400 m of sediment. Following the global geomorphic features map by Harris et al. (2014), the ARS is a 1,000 km spreading segment with a well-defined 830 km long and 30–70 km wide median valley. Its southern tip (Gríđargljúfur Gorge, Fig. 1) belongs to the extinct spreading center while being slow spreading (when the segment was active) and becoming the “fanning” extension. However, both sides of the valley are surrounded by abyssal hills and plains and situated within the Norwegian Basin. The valley rims lay 700 m below sea level at the southern tip near Iceland and deepen towards the north to reach a depth of 3,800 m in the Norwegian Basin.

The ARS is interesting from both a geological and biological point of view, as it is geomorphologically a rift valley, but biologically reminiscent of a canyon-like structure with steep walls and large bathymetric differences linking the upper slope of the GIF and the abyss of the Norwegian Sea. The heterogeneous environment in terms of substrate, hydrography, and depth is likely to support distinct faunal communities. The northern part of the ARS, being located in international waters in an area called “Banana hole” nested between the Norwegian, Icelandic, Faroe’s, and United Kingdom’s EEZs, is under consideration as a candidate for an Ecologically and Biologically Significant Area (EBSA) under the Convention on Biological Diversity by Norway, as it is in the area beyond the 200 nautical mile baseline area that Norway claims under its Continental Shelf Submission for the United Nations Convention for the Law of the Sea (Norwegian Petroleum Directorate, 2006). Its “fanning” extension is located in the Icelandic EEZ, and is not included in the Norwegian efforts of describing an EBSA area. The EBSA criteria provided by the CBD may thus create the prerequisites for a protection status of the region in the long term. VME indicator species as defined by the Food and Agriculture Organization of the United Nations criteria (FAO, 2009; ICES, 2020) can be modelled for the areas in the vicinity of mapped findings using species distribution models such as used by Burgos et al. (2020). Morato et al. (2018) and Morato et al. (2021), suggest these models are vastly improved by areas adding new data.

In this study, we present a description of the physical and biological components of the ARS based on data collated during the IceAGE3 expedition (Brix et al., 2020). As knowledge about depth zonation is rather scarce, our focus is more on the depth gradient than on community analysis. Specific objectives were to describe the area’s seafloor topography at 20–50 m resolution using multi-beam bathymetry. Furthermore, to analyze the epifaunal mega- and macrofauna composition of the ARS and its adjacent abyssal plain and infer its relationships to environmental variables. We expected the canyon-like topography of the ARS to promote a fauna with corresponding biological characteristics such as higher densities and diversity compared to the adjacent flat and sediment-dominated abyssal plain (De Leo et al., 2010; Riehl et al., 2020). In addition, we assumed that the physical environment, especially water mass and depth, had a major impact on community composition and distribution. In particular, we hypothesize that faunal communities shifted in relation to changes in environmental factors, notably depth, temperature, salinity, oxygen, and productivity, along the ARS. Finally, we discuss our findings in light of international agreements established to protect vulnerable marine ecosystems in the North Atlantic and Arctic oceans.

## Materials & Methods

### Sampling

During the IceAGE3 expedition (Icelandic marine Animals: Genetics and Ecology) on RV Sonne as SO276 (MerMet17-06) in June/July 2020, we investigated the central and southern sections of the ARS, referred to as Ægir Ridge ‘North’/‘N’ and Ægir Ridge ‘South’/’S’, using multibeam hydroacoustics, CTDs, an epibenthic sledge (EBS), and the remotely operated vehicle (ROV) Kiel 6000 (Figs. 2 and 3; Brix et al., 2020). The sampling design of the IceAGE project places a CTD into the center of each working area to link all benthic gear deployed with abiotic data from the water column is outlined in Brix et al. (2014). Abiotic variables analyzed from the CTD are temperature, salinity, and oxygen. In particular salinity and temperature are used as variables to define the water masses around Iceland to receive background information for biology (Brix & Svavarsson, 2010; Brix et al., 2018a; Brix et al., 2018b).

**Figure 4:**
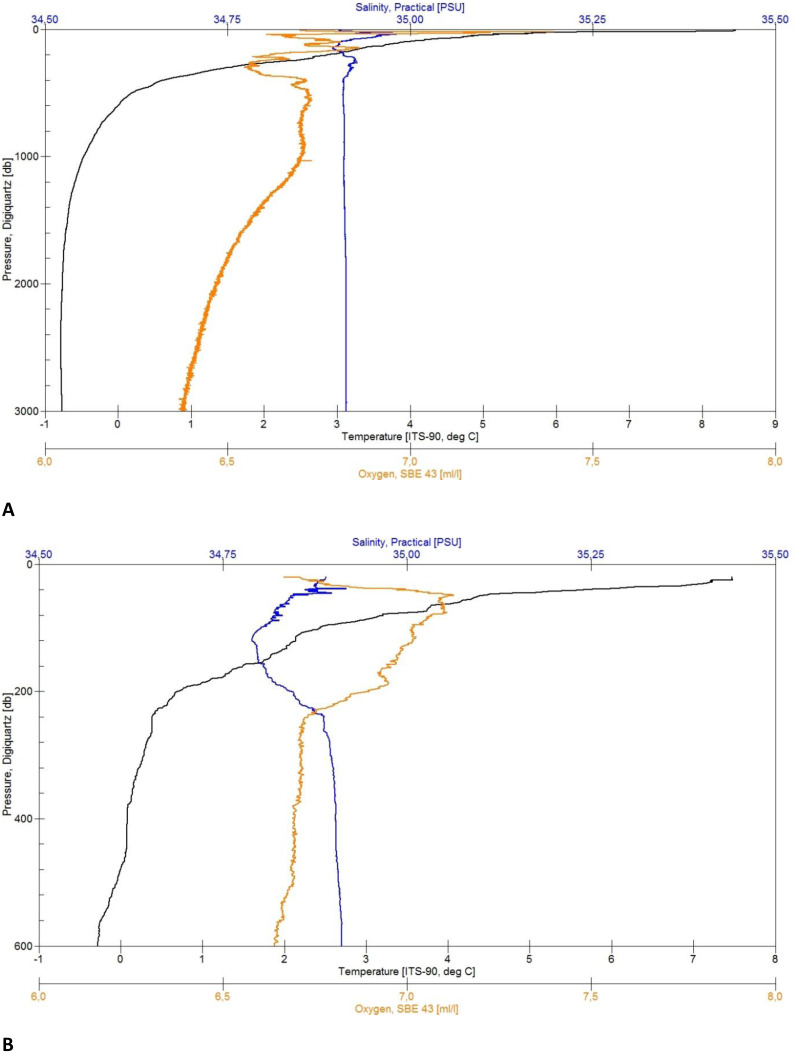
CTD plots. Environmental variables water column profiles of Station 013 (A) a deep, abyssal plain CTD location casting to 3,008 m depth and Station 043 (B) shallow profile, ridge top CTD location casting to 603 m depth.

### Bathymetry

Prior to sampling, multibeam bathymetry surveys of the areas were carried out using the RV Sonne’s hull mounted Kongsberg SIMRAD EM122 multibeam echo sounder system (MBES) to create high resolution maps of the sampling sites. This MBES operates at a frequency of 12 kHz and can therefore reach down to water depths of 12,000 m. With the automatic ping rate, varying with depth from 0.2–0.06 pings/second between −1,000 m and −4,000 m, a vessel speed of ∼8 kn during the surveys and swath widths of 90°–110° (depending on the data quality), bathymetric maps with a grid resolution of 20 m (at the shallower southern ridge end called Gríðargljúfur Gorge, Fig. 3) and 50 m (in the valley center called median valley between Ránarhryggur Ridge and Drafnarhryggur Ridge) were achieved (Fig. 2). The raw data point cloud was post-processed, *i.e.*, cleaned for outliers and gridded, with QPS Qimera 2.0 software. The processed data products were exported as interpolated grids to be visualized as maps with the open-source software QGIS 3.16.4.

### Oceanography

In total five CTD casts were deployed in the described area, ranging from 246–3,571 m in the maximum profile depth, which is represented by Fig. 4A for deep- and Fig. 4B for shallow profiles. The CTD stations visible on the map (Figs. 2 and 3) indicate the RV Sonne ship’s owned SBE 911plus CTD unit (Seabird Electronics). It was mounted approximately 1 m below the water sampler equipped with double conductivity, temperature, oxygen, chlorophyll (Chl), and turbidity sensors (Table 1). The water sampler was equipped with 24 × 10 l Niskin bottles that were electronically triggered to close at given depths on the upcast of the CTD profiles. The sampling rate of the sensors amounts to 24 Hz with 41 Bytes per scan. Data recording and Niskin bottle triggering was controlled with SEASAVE V7 software from a ship mounted computer. The GPS positions during each profile were logged from an NMEA-string of the RV Sonne. Each cast was processed by using SBE Data Processing to convert the binary (.hex) data to ASCII (.cnv) data.

**Table 1:** Water variables. Summary of environmental water column variables measured at the Ægir Ridge System by SBE 911 CTD used in this study.

**Station**	**Date**	**Position**		**Bottom**	**SBE911**								**Turbidity (NTU)**
		**Latitude**	**Longitude**	**Depth (m)**	**CTD Depth (m)**	**Temperature (°C)**	**Salinity (PSU)**	**O**_**2**_ **(ml/l)**	**max. O**_**2**_ **(ml/l)**		**max. Chl a (mg/m** ^**3**^ **)**		
13	28.06.2020	65°42.676N	002°57.153′W	3,341	3,008	−0.763	34.912	6.386	Depth (m)		Depth (m)		0.078
									26	7.200	26	2.097	
23	29.06.2020	65°55.471′N	003°33.628′W	3,692	3,571	−0.717	34.913	6.338	Depth (m)		Depth (m)		0.080
									30	7.400	44	1.600	
29	30.06.2020	66°10.152′N	004°21.613′W	3,423	3,403	−0.737	34.917	6.343	Depth (m)		Depth (m)		0.078
									42	7.165	42	1.420	
34	01.07.2020	66°03.138′N	003°59.896′W	3,675	3,010	−0.762	34.913	6.370	Depth (m)		Depth (m)		0.077
									42	7.059	29	1.314	
41	02.07.2020	65°16.296′N	007°56.559′W	1,413	246	0.764	34.878	6.775	Depth (m)		Depth (m)		0.081
									43	7.300	34	3.112	
43	03.07.2020	64°52.862′N	009°37.831′W	716	603	−0.291	34.911	6.633	Depth (m)		Depth (m)		0.080
									49	7.112	34	2.635	
62	06.07.2020	64°53.019′N	009°39.247′W	686	674	−0.342	34.911	6.613	Depth (m)		Depth (m)		0.085
									68	7.134	10	2.943	

The CTD data, as well as the plots (Figs. 4A and 4B) were untreated when used for first on board analyses of the sediment and further station work decisions. Depending on the weather conditions, some profiles started at a depth of approximately 20 m below surface for safety reasons (Fig. 4B). The quality control of the salinity values was done by taking water samples from the Niskin bottles during the upcast to analyze them in a land-based laboratory, using a Guildline 4800B Lab Salinometer. The samples were taken from the maximum depth and in homogenous water layers.

### Habitat characterization and megafaunal sampling by ROV

The ROV Kiel 6000 was deployed for three dives with a total of 26:08 h deployment time at the ARS (Table 2). For seafloor imagery (photos, video, and frame grabs of videos) ROV Kiel 6000 was equipped with two HDTV cameras (Sulis and ALPHA +) and two SD-color zoom video cameras OE14-366 MKII, the latter mounted on pan and tilt units. The footage of all of these were permanently recorded. Additionally, ROV Kiel 6000 is equipped with four black and white video observation cameras. Lighting for the video cameras is provided by two 400 W HMI SeaArc^®^2, two 70 W HID SeaArc^®^5000, and eight dimmable 250 W halogen SMulti-SeaLite^®^ lights. Additionally, two Alpha Cam lasers, 6.7 cm apart, were mounted parallel to the focal axis of the video camera to provide scale in images.

**Table 2:** ROV dives. Summary of ROV Kiel 6000 dive locations at the Ægir Ridge System as used in this study.

**Station**	**Dive No.**	**Date**	**Latitude**	**Longitude**	**Latitude**	**Longitude**	**Depth**	**Depth**
	**Kiel 6000**		**Start**	**Start**	**End**	**End**	**Start (m)**	**End (m)**
37	298	01.07.2020	66°03.170′N	003°59.914′W	66°03.684′N	004°01.952′W	3,700	3,419
54	299	04.07.2020	64°50.818′N	009°36.219′W	64°50.080′N	009°37.720′W	2,030	1,838
64	300	05.07.2020	64°52.180′N	009°37.471′W	64°52.835′N	009°37.548′W	1,411	740

ROV dive 37 investigated the abyssal plain in the central ARS median valley (Fig. 2) along a line from 3,700 m to 3,450 m depth (Fig. 2, Table 3). The ROV dives 54 (2,030 m to 1,700 m) and 64 (1,400 m to 740 m) investigated the southwestern end of the ARS along bathyal transects from the foot of the Grí ðargljúfur Gorge to its plateau called Skjaldargrunn Bank (Fig. 3). Digital still images taken during the dives were used for the identification of selected prominent or abundant megafauna and the identification followed the best practice for the use of open nomenclature signs to image-based faunal analyses (Horton et al., 2021) (Table 4). Observed species were assessed as to their VME indicator status following NEAFC 2014 and the latest lists of VME indicators defined by the ICES Working group on Deep-Water Ecology (WGDEC, ICES 2019, ICES, 2020).

**Table 3:** EBS stations. Summary of epibenthic sledge stations conducted during the IceAGE3 (MerMet 17-6/SO276) expedition along the Ægir Ridge System as used in this study. Information includes area, latitude and longitude (in degree), depth (m), time and trawling distance.

**Station**	**Date**	**Latitude start**	**Longitute start**	**Depth start (m)**	**Trawling distance (m)**
16	28.06.2020	65°43.552′N	002°58.158′W	3,363	576
26	29.06.2020	65°56.122′N	003°31.727′W	3,702	648
30	30.06.2020	66°09.596′N	004°20.937′W	3,467	612
39	01.07.2020	66°02.682′N	004°00.570′W	3,678	486
47	03.07.2020	64°48.515′N	009°31.816′W	2,289	540
55	04.07.2020	64°53.509′N	009°38.047′W	681	360
61	05.07.2020	64°52.979′N	009°39.289′W	686	198

**Table 4:** Taxa. Selected, noticeable megafauna elements and their depth occurrences on *in-situ* ROV imagery on the Ægir Ridge used in this study. Phyla and VME indicator taxa marked in bold.

**Phylum class**	**Order**	**Family**	**Lower rank**	**Label in habitat schematics**	**Depth min. (m)**	**Depth max. (m)**
**Porifera**						
Demospongiae	Axinellida	**Axinellidae**	***Phakellia*** **sp. indet**	*Phakellia*	1,230	2,000
Demospongiae	Poecilosclerida	Cladorhizidae	*Asbestopluma furcata*	*Asbestopluma*	1,220	2,000
Demospongiae	Poecilosclerida	Cladorhizidae	*Chondrocladia grandis*	*Chondrocladia*	750	900
Demospongiae	Poecilosclerida	**Geodiidae**	***Geodia hentscheli***	*Geodia*	1,030	1,970
Demospongiae	Poecilosclerida	**Coelosphaeridae**	***Lissodendoryx complicata***	*Lissodendoryx*	1,850	1,950
Hexactinellida	Lyssacinosida	**Rossellidae**	***Caulophacus arcticus***	*Caulophacus*	3,530	3,700
Hexactinellida	Lyssacinosida	**Rossellidae**	***Schaudinnia rosea***	*Schaudinnia*	850	1,970
**Mollusca**						
Chephalopoda	Octopoda	Opisthotheuthidae	*Grimpotheuthis* sp. indet			3,600
Gastropoda	Neogastropoda	indet	Neogastropod sp. indet.			1,930
**Echinodermata**						
Asteroidea	Velatidae	Pterasteridae	*Hymenaster* sp. indet	*Hymenaster*	1,100	1,950
Crinoidea	Comatulida		Comatulid sp. indet	Crinoid	750	1,220
Ophiuroidea	Euryalida	Gorgonocephalinae	*Gorgonocephalus* sp. indet		750	1,300
**Echiuroidea**			Echiuroid indet	Echiuroid	1,450	3,700
**Arthropoda**						
Pycnogonida	Pantopoda	indet	Pycnogonid sp. indet			3,700
		Colossendeidae	*Colossendeis* sp. indet		840	1,330
Malacostraca	Decapoda	Bythocaridae	*Bythocaris* cf. *leucopis*	*Bythocaris*	1,900	3,700
	Amphipoda	Calliopiidae	Calliopiid sp. indet	Calliopiid	1,900	3,700
**Cnidaria**						
Anthozoa	Alcyonacea	**Nephtheidae**	***Drifa glomerata***	Soft coral	750	1,450
	Alcyonacea	**Nephtheidae**	***Gersemia*** **sp.**	Soft coral	1,340	2,000
	Actinaria	Actinostolidae	Actinostolid sp. A indet	Actinostolid	3,530	3,530
	Actinaria	Actinostolidae	Actinostolid sp. B indet	Actinostolid	1,930	3,680
	Actinaria	Actinostolidae	Actinostolid sp. C indet	Actinostolid	750	820

For horizontal megafauna zonation analysis, ROV footage was reviewed with the deepest and shallowest occurrences of selected taxa extracted (see Table 4). Additionally, five frame grabs per 25 m of depth were randomly selected from the ROV videos to assess presence and dominance of the selected taxa for the habitat schematics. This process involved extracting screen grabs from each depth zone, ascertained by the corresponding metadata files generated by the ROV, at 10 s intervals, assigning them a randomly generated number and then generating random numbers to select the corresponding images. This was done to eliminate observer bias. No individual abundance numbers of taxa per defined area analysis was done as the distance defining lasers were not on throughout the dives.

No statistical analyses could be conducted, because of the lack of replicates at the three sites.

### Taxonomic identifications of megafauna from ROV *in-situ* images

Taxonomic identification of megafaunal groups were made to the lowest taxonomic rank possible, from images obtained with the ROV, using the authors’ taxonomic expertise and recent revisions of specific groups (*e.g.*, on sponges, Cárdenas et al., 2013; Hestetun, Tompkins-Macdonald & Rapp, 2017) of the bathyal fauna of the boreo-Arctic region. When a particular taxon could not be confidently identified to species level, higher taxonomic ranks combined with open nomenclature signs (*e.g.*, ‘sp.’, ‘stet.’, ‘indet.’) were implemented following the standards proposed by Horton et al. (2021).

### Macrofauna sampling by epibenthic sledge (EBS)

The macrofauna were examined with an EBS (Table 2) with just an epibenthic sampling unit such as is in the EBS by Rothlisberg & Percy (1977) and corresponding to the epi-samplers of related EBS types, which have an epibenthic and a suprabenthic sample unit (Brandt & Barthel, 1995; Brenke 2005; Brandt et al., 2013). We deployed one EBS at each depth, where it was possible to deploy an EBS. This is a trawled gear and can only be deployed where there is enough space/room for it on the seafloor. Thus, the macrofauna data cannot cover the “wall”, which has been sampled/observed via ROV dives. The aim was to deploy the EBS on each side of the Æegir Ride in the north and on the shallower part and deeper parts in the south. A detailed description of the sampling procedures is given in Brix et al. (2020). In total (both, North and South samples as indicated on the overview map in Fig. 1) seven EBS were deployed in the ARS area ranging in depth from 681 m to 3,702 m (Table 3). Three stations were taken from the bottom of the median valley (stations 26, 39, and 47) and four stations from shallower parts (Ránarhryggur and Skjaldargrunn; Table 3). Trawling distance (d) was standardized to a trawled distance of 1,000 m for calculation of 1,000 m^2^ sampled seabed area, as the epibenthic sample unit is 1 m wide. We therefore used the following formula: d = (V_1_ × T_1_) + (V_2_ × T_2_) + (V_3_ × T_3_) (V_1_: ship velocity during trawling; T_1_: trawling time; V_2_: ship velocity during haul; T_2_: haul time (sled off bottom), V_3_: winch velocity; T_3_: haul time [sled off bottom]). As soon as the EBS arrived on deck, the cod end was retrieved and immediately taken to the cold room (+4 °C). The sample processing mostly followed protocols for a cold chain that enable later molecular analyses (Riehl et al., 2014). Specimens visible to the naked eye were picked from the bulk sample, photographically documented, and separately fixed (RNAlater and undenaturated 96% ethanol) or frozen for genetic, genomic or biochemical analysis. The remaining sample was carefully elutriated in pre-chilled filtered seawater, then sieved through a 300-µm mesh, fixed in pre-chilled (−20 °C) 96% denatured ethanol and stored at −20 °C for at least 48 h. Sorting of the samples began on board and was continued in the Senckenberg laboratory (DZMB, Hamburg) and due to pandemic restrictions finished in “home office”. Specimens were assigned to major taxonomic units (phylum, class, order level).

### Analysis of macrofaunal taxa

Comparisons of macrofaunal abundances obtained from the EBS were made based on density data. Therefore, total abundances were standardized to individuals per 1,000 m^2^ seabed area.

PRIMER v6 (Clarke & Gorley, 2006) was used for the multivariate statistical analysis. A one-way Analysis of Variance (ANOVA) was used to test for differences in macrofaunal densities between stations using SigmaPlot Version 12.5. To test for homogeneity of variance Levene’s test was used, while we used Shapiro–Wilk test to test for normality. In case assumption of normality and homogeneity of variance were not met, a non-parametric Kruskal-Wallis test was applied. Sample size between EBS deployments may differ (see Table 3), and thus samples provide arguably only semiquantitative data (Kaiser & Brenke, 2016), To assess differences in macrofauna community composition between samples, Bray–Curtis similarities were calculated for non-transformed relative abundance (percentage) data of total macrofauna obtaining a similarity matrix (Clarke & Gorley, 2006). Relative abundances for the multivariate analysis were used to account for this. Non-metric multi-dimensional scaling (nMDS) implemented in PRIMER v6 was used to visualize differences between stations and depth.

Relationships between macrofaunal densities, environmental factors (T, S and O_2_), and depth were explored using Spearman rank correlation coefficient in Sigmaplot 12.0 for total macrofauna. Furthermore, relationships between macrofaunal composition and environmental variables were explored using the Biota and Environment matching (BIO-ENV) procedure based on normalized Euclidean distance. All parameters were previously tested for collinearity using a draftsman plot calculated in Primer v6.

## Results

### Bathymetry

The two regions of the ARS investigated and mapped during the IceAGE3 expedition present a contrasted ridge morphology. In the central region (named North) near 66° N, the ARS is a ∼27 km wide axial valley at a depth of 3,700 m–3,800 m. The eastern and western valley walls Ránarhryggur Ridge and Drafnarhryggur Ridge have gentle slopes (less than 15°). The valley is bound by two broad shoulders (16–18 km wide), a relict of a split volcanic ridge. They culminate at a depth of 3,000 m and 2,000 m for the western and eastern shoulder, respectively. These shoulders separate the median valley from the surrounding abyssal plain at ∼3,400 m. South, at Gríðargljúfur Gorge (southern end of the ridge), the axial valley is wider (67 km) with a shallower valley floor laying at 2,300 m–2,600 m below sea level. The section map (at ∼64.8° N) shows that the valley walls become steeper (1–60°). In this region, which lays between 700 and 1,100 m below sea level, there is no shoulder between the valley and the surrounding seafloor.

**Figure 5:**
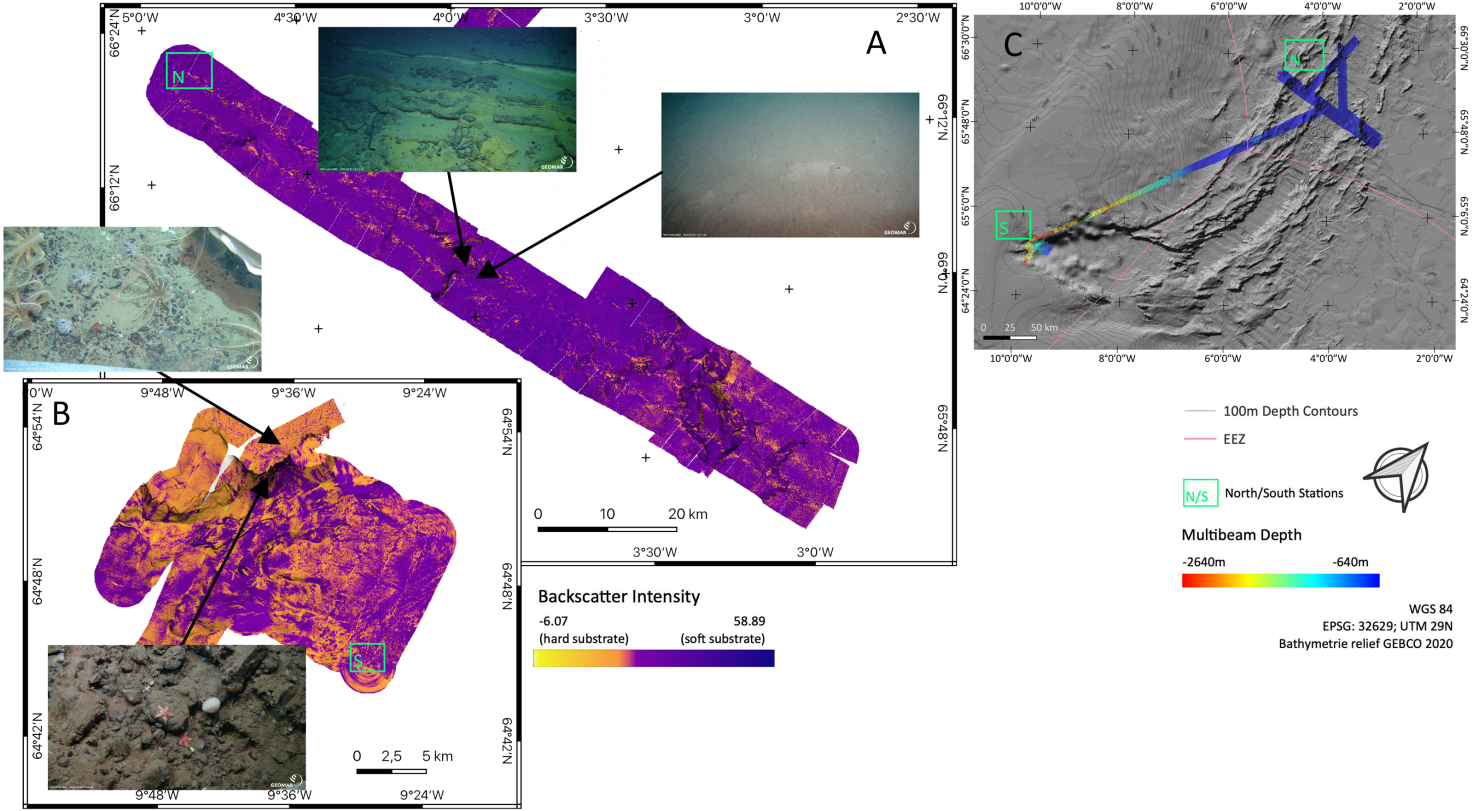
Backscatter data. Detailed maps of sediment backscatter information maps of sampling areas (A) ‘North’ and (B) ‘South’ at the Ægir Ridge including an overview map as orientation (C). *In-situ* images show representative hard and soft substrates observed. Maps produced with software QGIS 3.16.4.

Backscatter analyses (Fig. 5) in both sampling areas (North and South as indicated in Fig. 5C) revealed that the seafloor along and across the ARS does not have a homogeneous acoustic characterization and systematic variations are evidenced. The valley floor has always a lower backscatter amplitude than the surrounding abyssal plain. The shallowest southern end of the ridge has a higher backscatter amplitude than the deeper central section of the ridge. The highest amplitude is found on a wall where the depth gradient exceeds 30°. Variations in the intensity of the acoustic signal, reflect change in the substrate hardness. Low backscatter amplitudes are attributed to soft substrate and high backscatter to hard substrate. Correlation between backscatter signal and substrate is observed from ROV footage, especially between the between north and south region. Images of the seafloor show that rock fragment, mostly dark grey and black, up to few decimeters in diameter are predominant on the southern section of the ridge. Similarly, steeper slope on the valley wall exposed larger blocks or compacted sediment layers.

### Environmental parameters–CTD

Each of the CTD profiles shows water masses with different properties of temperature and salinity (Figs. 4A and 4B) in the upper layer (0–600 m), creating an inhomogeneous mixed layer with a relatively strong thermocline. The range in temperature (*e.g.*, Station 13: 8 to 0 °C) and salinity (35.05 to 34.89 PSU) shows the impact of the colder and denser Artic-origin water from the south and Atlantic-origin water from the north (Puerta et al., 2020). The 0 °C point was reached after 600 m and is generally considered as an indicator for Artic-water masses (Semper et al., 2019). At this depth down to the bottom the change in temperature (*e.g.*, Station 34: 0 to −0.77 °C) and salinity (34.91 to 34.92 PSU) slightly decreases, developing a more homogenous water mass. The minimum water temperature for each profile was reached at a depth of 1,500 m (*e.g.*, Station 29: −0.77 °C) turning into a homogenous water mass. The minimum salinity value was found in the described upper mixed layer (*e.g.*, Station 43: 34.78 PSU). The change in salinity decreases with depth and reaches a constant level after 1,800 m (*e.g.*, overall: 34.88 to 34.92 PSU).

Although some variables were strongly correlated (Spearman rank correlation, rho > 0.9), in particular salinity, oxygen, and chlorophyll, we kept all of them in the analysis, since the combination of water mass properties has been identified as important to explain differences in benthic community structure in the Nordic seas/North Atlantic (Weisshappel & Svavarsson, 1998; Puerta et al., 2020; Roberts et al., 2018).

The maximum oxygen value was located in the upper 70 m (*e.g.*, 7.06 to 7.40 ml/l). The effect of mixing water masses shows in the upper 500 m with the change of oxygen (*e.g.*, station 013: 7.20 to 6.55 ml/l) until it constantly decreases with the depth (Fig. 4A).

### Megafauna based on ROV imagery

**Figure 6:**
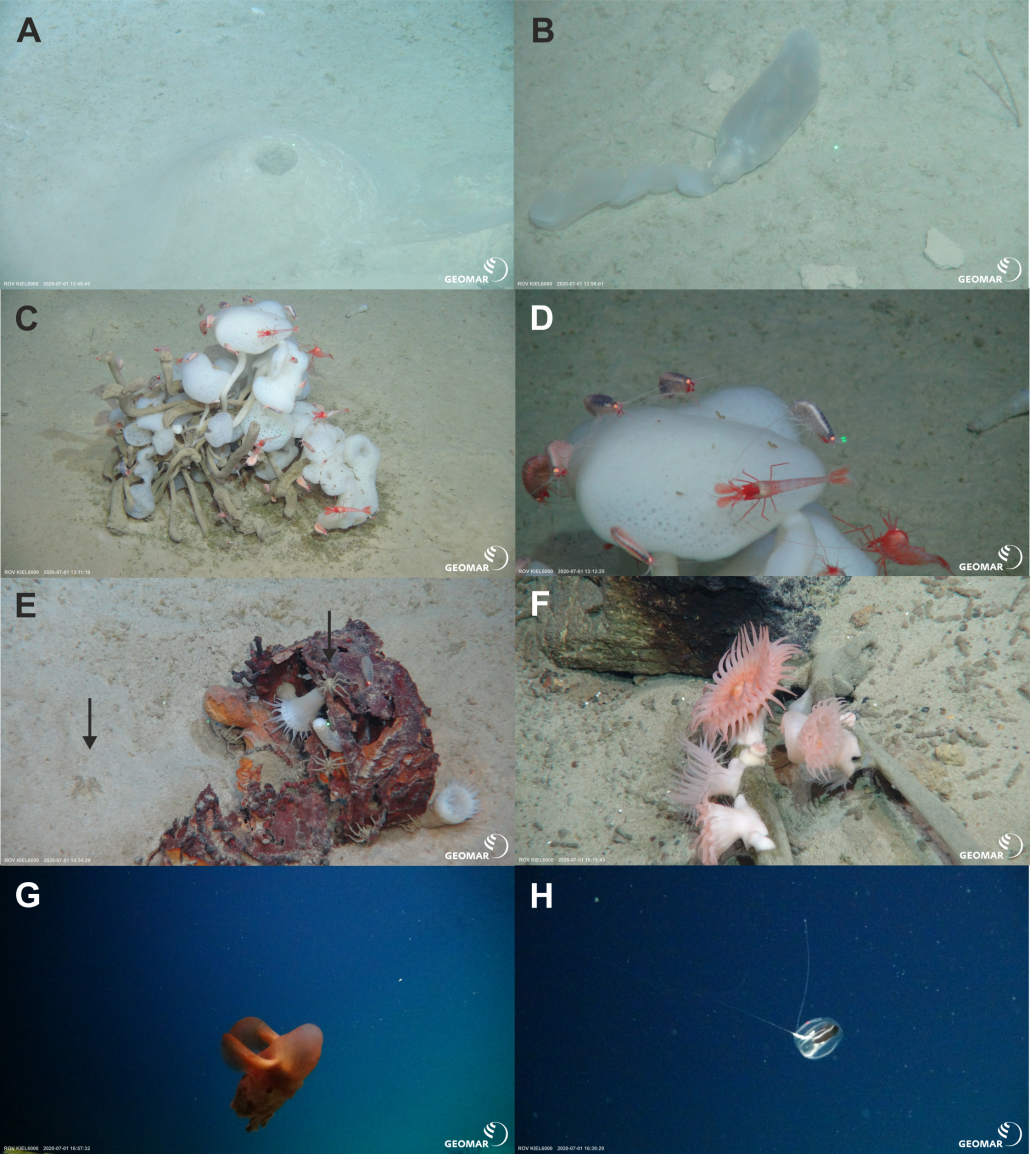
Megafaunal elements. *In-situ* images of characteristic megafauna of the Ægir Ridge at 3,400–3,700 m. (A) Echiurid mount. (B) Echiurid. (C & D) Glass sponge *Caulophacus arcticus* with caridean decapods and amphipod peracarids. (E) Actinostolid sp. A anemones and Pycnogonida sp. seaspiders (arrows). (F) Actinostolid sp. B anemones on *Caulophacus* stalks. (G) Dumbo octopus, *Grimpotheuthis* sp. (H) Ctenophore. Image source credits: GEOMAR/Senckenberg.

**Figure 7:**
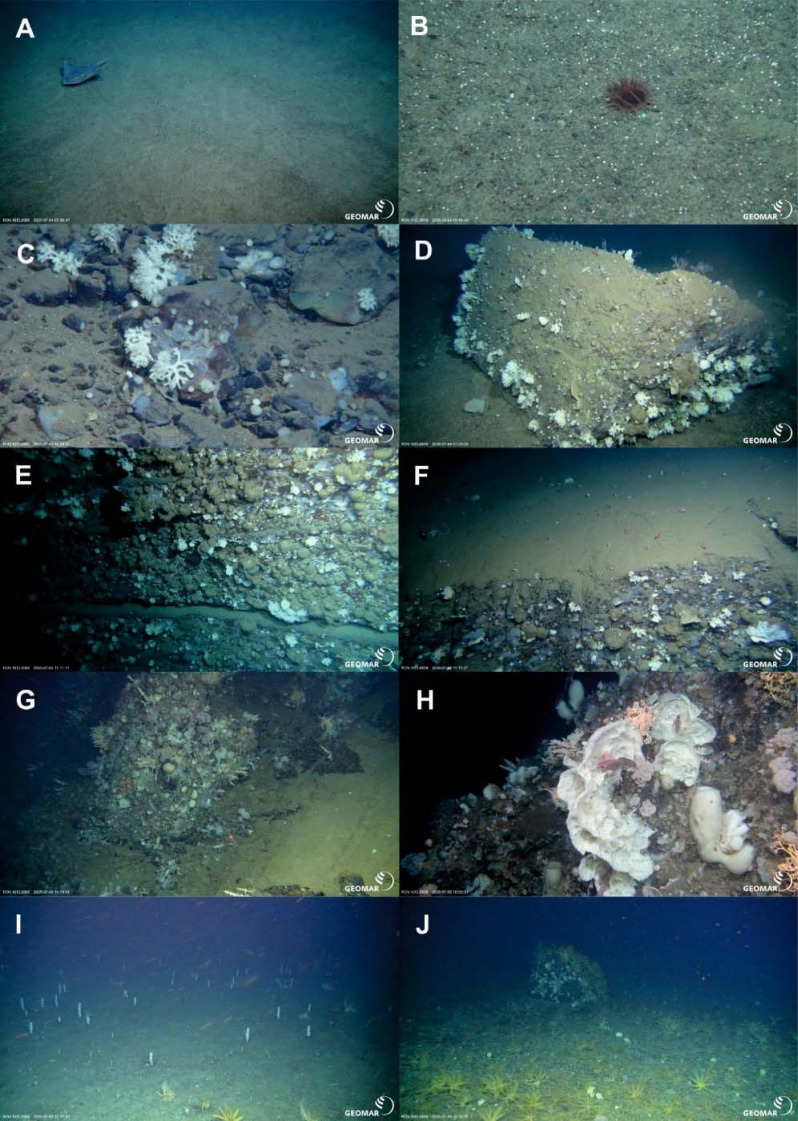
Geomorphology. *In-situ* images of geomorphology types of the Ægir Ridge at 2,000 m–600 m. (A & B) Coarse soft sediments. (C) Cobbles and pebbles on soft sediment (D) Sediment block on soft sediment. (E) Steep consolidated sediment layers. (F) Sedimented, flat steps of consolidated sediment layers. (G & H) Steep hard rock outcrops. (I & J) Flattened ridge top with sedimented areas and sediment blocks. Image source credits: GEOMAR/Senckenberg.

**Figure 8:**
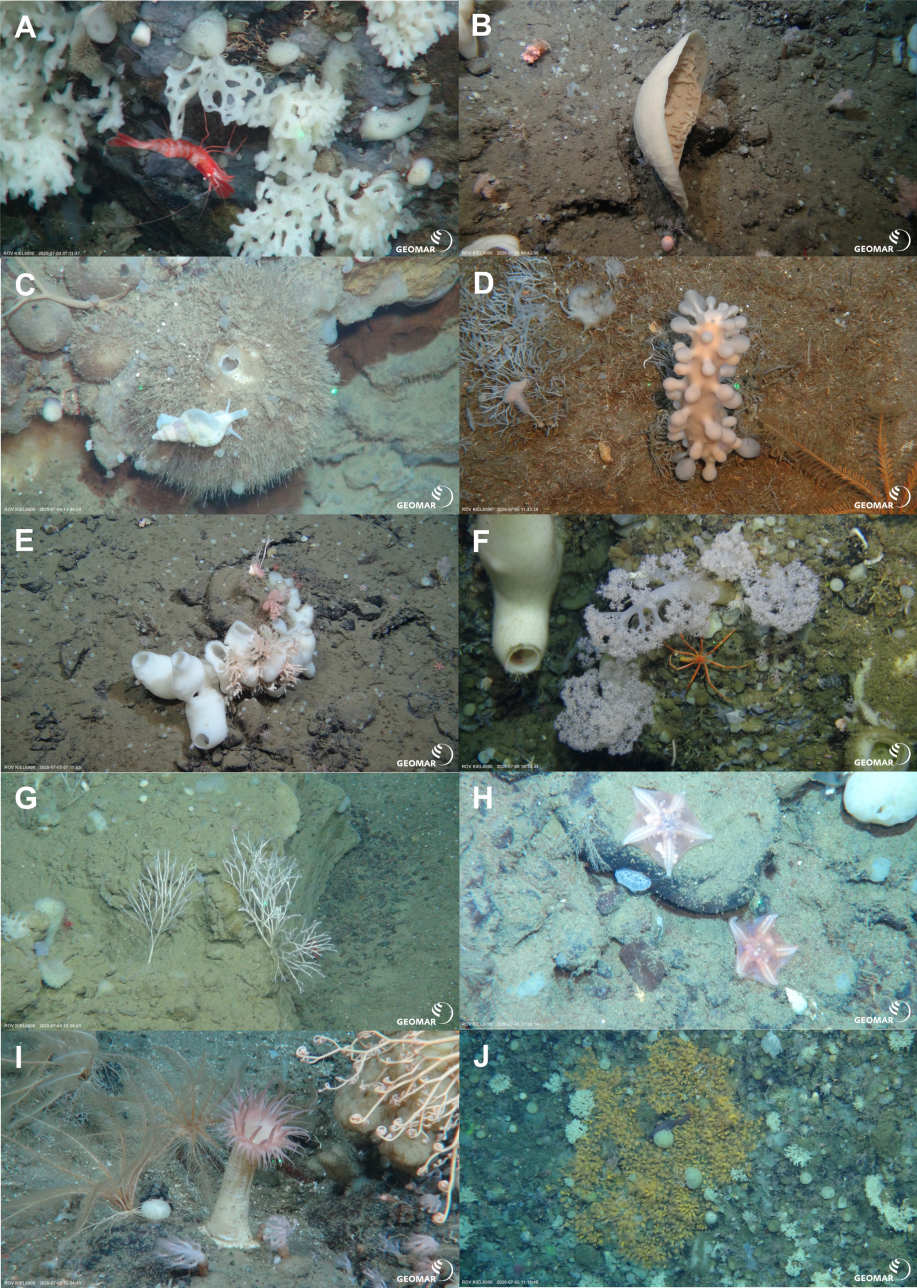
Characteristic megafauna. *In-situ* images of characteristic megafauna of the Ægir Ridge at 2,000–600 m. (A) Demosponge *Lissodendoryx* (*Lissodendoryx*) *complicata* with caridean decapod. (B) Flabellate demosponge *Phakellia* sp. indet. (C) The tetractinellid sponge *Geodia hentscheli* with neogastropod; (D) Carnivorous sponge *Chondrocladia* (*Chondrocladia*) *grandis.* (E) Glass sponge *Schaudinnia rosea* (F) Soft coral with *Colossendeis* sp. seaspider. (G) Carnivorous sponge *Asbestopluma* (*Asbestopluma*) *furcata*. (H) Pterasterid starfish *Hymenaster* sp. (I) Commatulid crinoids, basket star *Gorgonocephalus* sp. and actinostolid sp. C anemone. (J) Aggregations of demosponges and zoanthid cnidarians. Image source credits: GEOMAR/Senckenberg.

**Figure 9:**
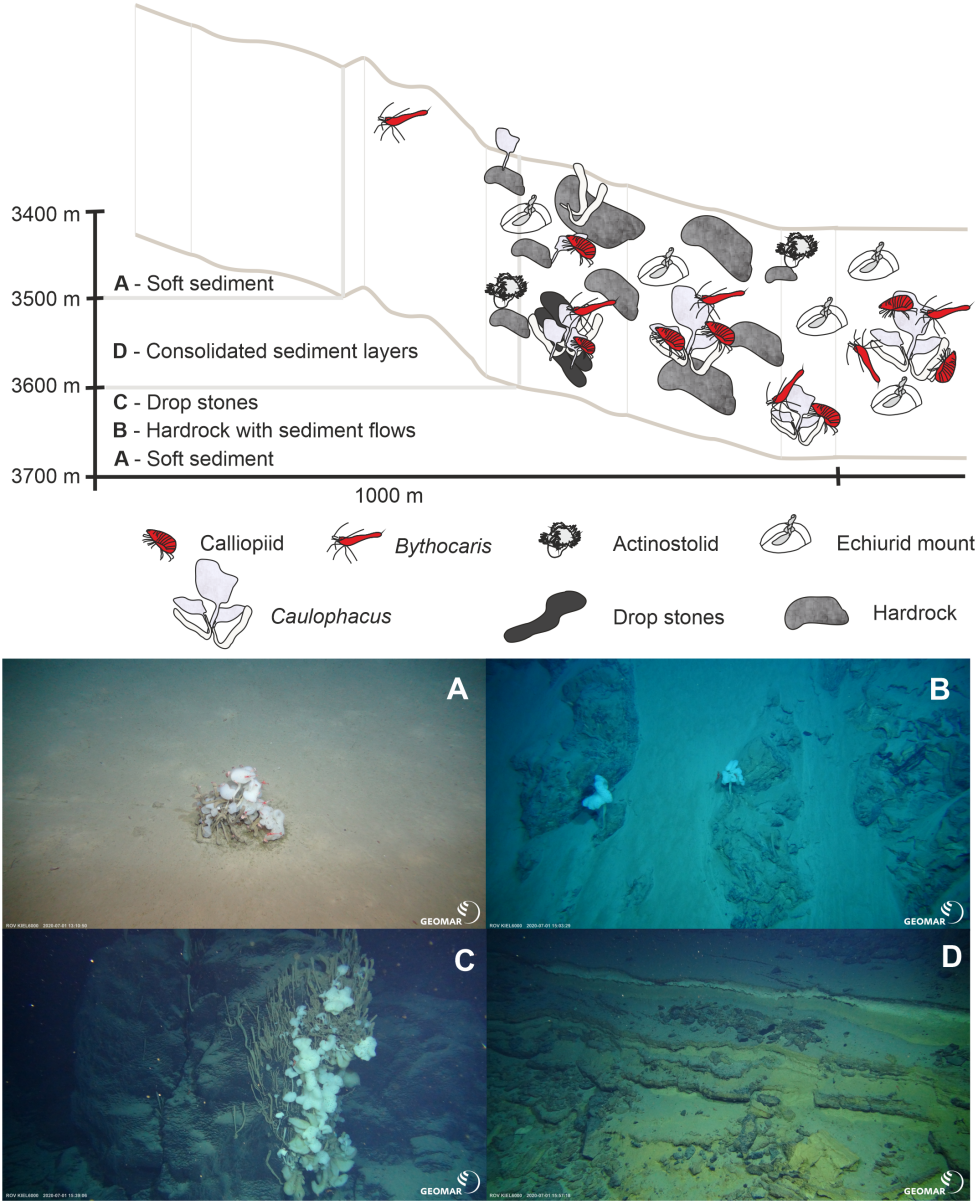
Schematic 1. The Ægir Ridge System at sampling area ‘N’: (A) Habitat schematic for the Ægir Ridge System sampling area ‘N’. *In-situ* images represent: (B) Soft sediment. (C) Hardrock with sediment flows. (D) Drop stones. (E) Consolidated sediment layers. Image source credits: BAS/Senckenberg.

During three ROV Kiel 6000 dives, *in-situ* video footage was taken at three sites with different depth ranges on the ARS and its adjacent abyssal plain and rise (Table 4, Figs. 2, 6, 7 and 8). The deepest ROV transect at station 37 (3,700 m to 3,450 m) from the sedimented plain in the median valley towards the rise of the ridge (Ránarhryggur) showed a highly sedimented habitat with sparse rocky outcrops in the abyssal plain area (Fig. 9). The sedimented areas showed Lebensspuren, including echiuroid mounts (Figs. 6A, 6B), irregular presences of burrowing cerianthid anemones, stalked crinoids (currently on the FAO NW Atlantic VME indicator taxa list), and the occasional rocks colonized by the glass sponge *Caulophacus arcticus* (Hansen 1885) (Figs. 6C, 6D). The *C. arcticus* colonies provide a habitat for aggregations of caridean shrimp (*Bythocaris* cf. *leucopis* GO Sars 1879) and amphipod species of different size classes, most prominently large amphipods from the family Calliopiidae, which are at present stage not identified as any described species and need further taxonomic attention (Fig. 6D). Ascending along the rise, the habitats changed from a sedimented seafloor to an area of hard rock outcrops separated by sediment flows, (Fig. 9C) to steep, consolidated sediment layers (Fig. 9D) and areas with drop stones, ending in a further sedimented area on the upper rise. The hard rock outcrops surrounded by sediment flows were colonized by occasional, single specimens of *C. arcticus*, while some drop stones were covered by dense colonies of this sponge. The consolidated sediment layers appeared bare of megafauna.

**Figure 10:**
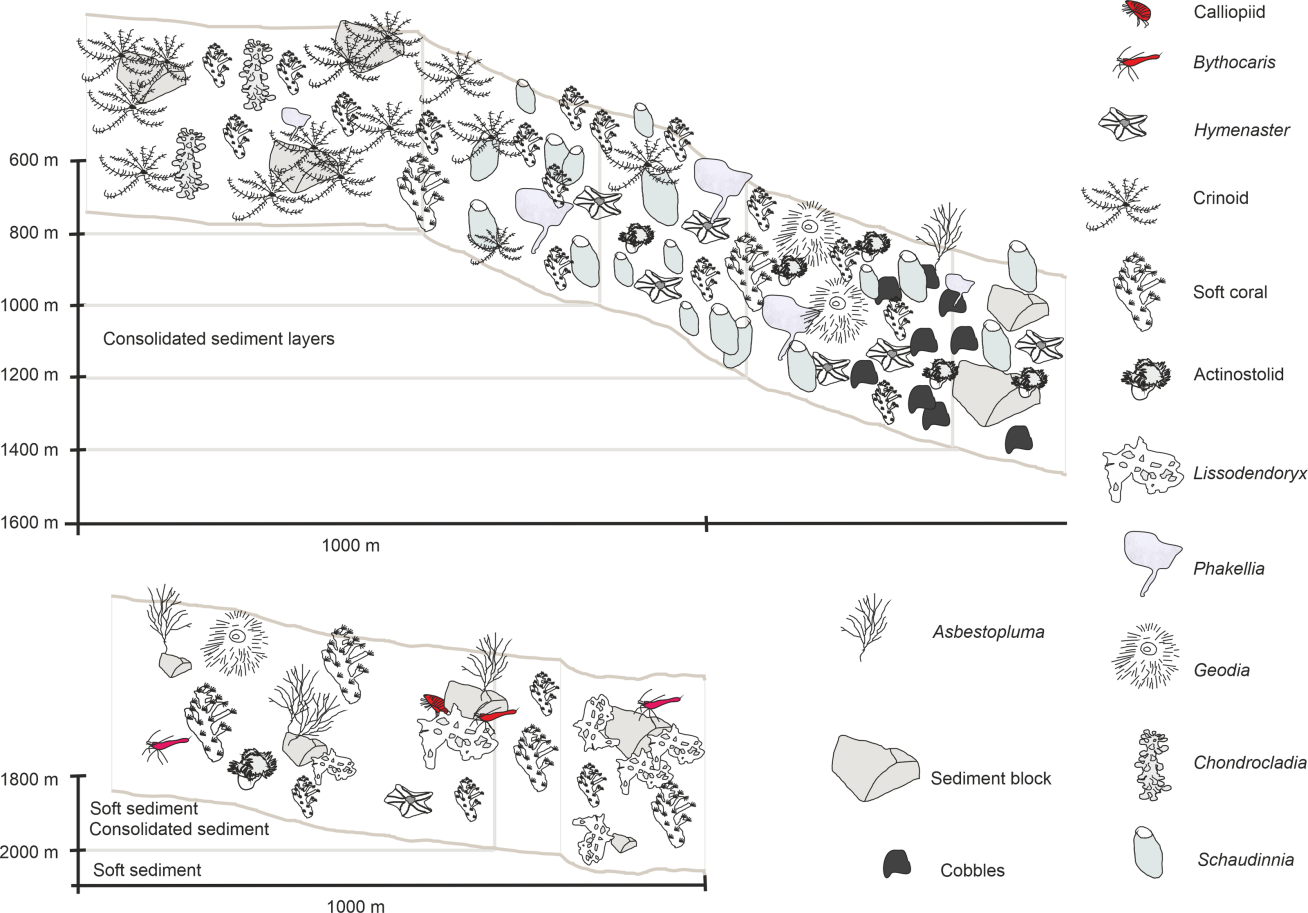
Schematic 2. Habitat schematic for the Ægir Ridge System in sampling area ‘S’. (A) Based on ROV Kiel 6000 dive 300. (B) Based on ROV Kiel 6000 dive 299.

The two ROV dives in Gríðargljúfur Gorge (southwestern end of the ARS) and the plateau Skjaldargrunn Bank started at the foot of the ridge structure in a flat, sediment covered bathyal area at 2,030 m depth and ended on the top edge of the ridge plateau (Skjaldargrunn Bank) at 740 m (Figs. 3, 7, 8 and 10; Table 4). The second ROV track at station 54, ascending from 2,030 m to 1,700 m depth, started in coarse sedimented areas which were interrupted intermittently by sediment blocks of various sizes, providing diverse habitats for epifaunal taxa, as well as cobbles and pebbles (Figs. 7A–7D). These hard rock surfaces were frequently colonized by various species of sponges, including the carnivorous sponge *Asbestopluma (Asbestopluma) furcata* Lundbeck, 1905 (Fig. 8G), with the regular presence of crustaceans, gastropods, and echinoderms. The sedimented areas were colonized by soft corals, sponges, and epifaunal pterasterid starfish. Closer to the steeper foot area of the ridge, consolidated sediment layers, densely colonized by sponges were seen (Figs. 7E, 7F). The third survey at station 64 ascended from the steep part of the ridge foot in 1,400 m to the ridge top (740 m) (Table 4, Fig. 10). The sediment layer became thinner to non-existent with increased steepness of slope (*i.e.*, a thick sediment layer at the bottom, only rocks and no sediment at the top, just rocks, see Fig. 5). Frequent steep hard rock outcrops and walls alternated with less steep areas, which then had thin sediment covers (Figs. 7G, 7H). These hard rock structures were densely covered by habitat forming sponges of different morphospecies including demosponges belonging to the families Cladorhizidae and Coelosphaeridae, as well as hexactinellids of the family Rossellidae. These sponges provided diverse habitats for aggregations of cnidarians, molluscs, pycnogonids, crustaceans, and echinoderms (Table 4). The flattened top of the ridge (Figs. 7I, 7J) was densely covered by comatulid crinoids, actinostolid actinians and sponges like the “club-shaped” *Chondrocladia (C.) grandis* (Verrill, 1879) (Fig. 8D).

The habitat heterogeneity and dominant megafaunal elements are characterized in schematics to visualize the change of sediment structure and faunal zonation along a depth gradient (Figs. 9 and 10). The first and last depth appearance of the dominant megafauna, as well as their status as VME indicators for NEAFC (ICES, 2020), was noted (Table 4) and indicates zonation of megafaunal communities along depth. The rossellid sponge *Caulophacus arcticus* was only observed in depths of 3,530 m–3,700 m, while the associated crustaceans were also observed on a different species of sponge until 1,900 m depth. Echiurid mounts were present in soft sedimented areas from 1,450 m to 3,700 m depth. The nephtheid soft coral *Gersemia* sp. was present at 1,340 m to 2,000 m, white *Drifa glomerata* (Verrill, 1869) was present at 750 m to 1,450 m but was most dense at 1,000 m to 1,250 m. The sponges *Phakellia* sp. indet and *A. (A) furcata* were present at 1,220‘ m to 2,000 m, while the rossellid *Schaudinnia rosea* (Fristedt 1887) was observed from 850 m to 1,970 m. The carnivorous *C.* (*C.*) *grandis* first appeared at 900 m depth and was more frequent on the ridge top at 750 m depth. The large, multiarmed ophiuroid *Gorgonocephalus* sp. indet and the comatulid sp. indet crinoids, were first recorded on the upslope ROV dive around 1,250 m depth and were present to the ridge top at 750 m depth, where the comatulids showed their highest densities.

The ARS valley abyssal plain hosted VME indicator species (Figs. 6–10, Table 4) especially sponges indicating habitat like “deep sea sponge aggregations” as well as “sediment emergent fauna”. In the sedimented areas Cerianthidae were observed, they are listed as VME indicators of “tube-dwelling anemone” patches (Fig. 7B). The gentle and steep slopes of the southwestern ARS of hard-bottom and soft-sedimented substrate showed a variety of VME taxa, including the soft coral family Nephtheidae, with the species *Gersemia* sp. abundant at 1,340 m to 2,000 m depth indicating “cauliflower coral fields”. The hard bottom areas, especially on steep slopes, provided habitat to dense, species rich deep-sea sponge aggregations. Habitat forming species like the rossellid and poecilosclerid sponges hosted dense communities and harboring numerous fauna. While not a VME indicator taxon, high species richness and abundance of carnivorous sponge taxa including *A.* (*A.*) *furcata,* and *C.* (*C*.) *grandis* was noted at all depths investigated by ROV surveys at the southwestern ARS site, which suggests the presence of rich supra-benthic food resources, like swimming crustacean taxa, *e.g.*, Amphipoda, Copepoda, or Isopoda (Table S1).

### Macrofauna based on EBS collections

**Figure 11:**
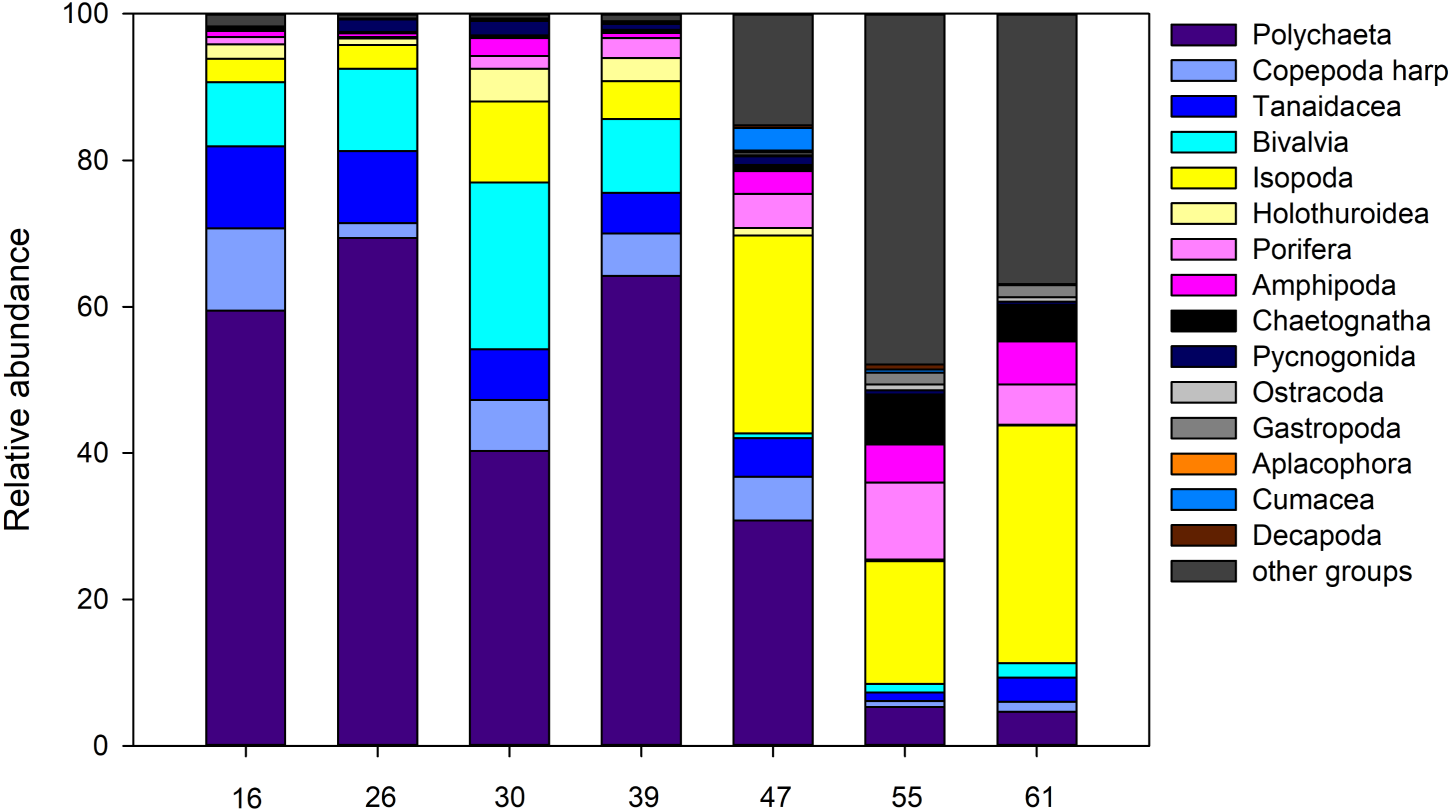
Abundance. Relative abundance (%) of macrofauna collected from the Ægir Ridge by means of an epibenthic sledge.

For the entire macrofauna there seems to be a slight trend towards higher densities at the shallow sites (stations 55 and 61) compared to the deeper ones (Table S1). However, this is largely due to the high proportion of calanoid copepods at epibenthic depths at these stations, which make up between 53.0% and 75.8% of the total macrofauna there (Fig. 11). The mechanism of the EBS, which closes the sample unit as soon as seafloor contact is lost, and remains closed through the water column, precludes collection in the pelagic zone. When removing calanoids from the analysis, macrofauna densities at the deeper part of the ARS (stations, 26, 39, and 47) were significantly higher compared to the other stations (one-way ANOVA, F1,6 = 35.6; *p* < 0.002). Overall, Polychaeta was the most dominant taxon with 41.9% of the total macrofauna, followed by the Isopoda (14.2%), Ophiuroidea (9.0%), Bivalvia (7.1%) and Tanaidacea (6.2%). Yet, the relative abundance of each taxon varied between stations and depth. While polychaetes showed high relative abundances at the deep stations, where they contributed between 30.8% and 69.4% to total macrofauna, they were only poorly represented at the two shallow stations (4.7–5.3%). Here, isopods and ophiuroids were more dominant. Differences in macrofaunal densities could not be related to any of the measured environmental, also water mass defining variables or depth (Spearman rank; *p* > 0.05). For polychaetes, however, depth was identified as the most important factor explaining density variation densities (Spearman rank; rho = 0.89, *p* = 0.0000002), whereas the remaining factors were revealed as not significant (Spearman rank; *p* > 0.05).

**Figure 12:**
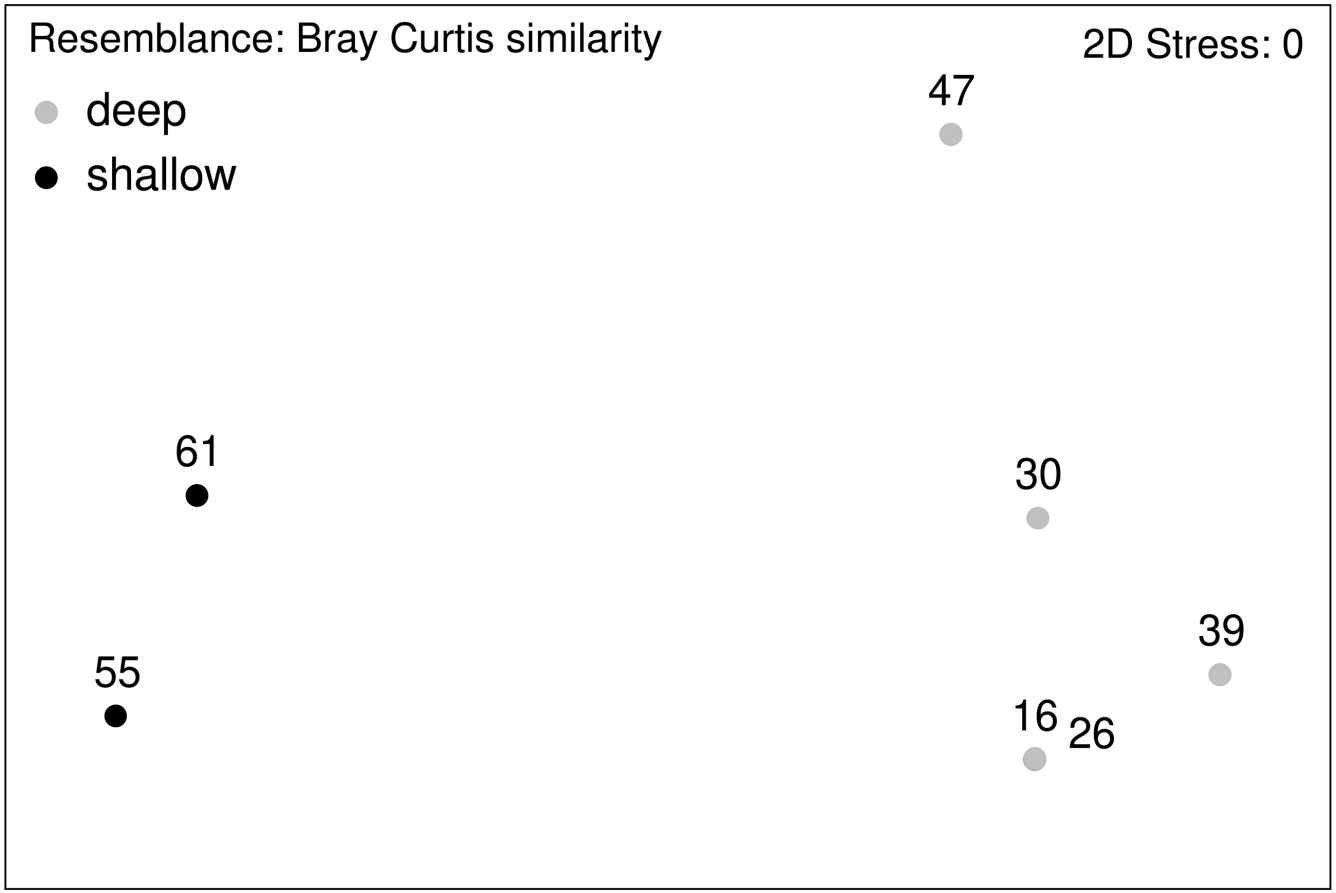
nMDS plot. Macrofauna assemblage analyses of EBS samples (nMDS plots).

Community analysis of the total macrofauna, as visualized by nMDS, showed a clear division between shallow parts of the ARS (Stations 55 and 61) and the deeper ones (Fig. 12). Since calanoid copepods made up a large fraction at these stations, analyses were repeated without them, but the same pattern remained. The correlated environmental variables T, S, and O_2_ gave evidence for the presence of water masses of North Atlantic origin at the shallower stations, while the deep-sea stations were under Arctic water mass influence. These water mass differences and especially the presence of their associated mesozooplankton community is likely have an effect on the benthic community composition.

## Discussion

The deep-sea floor is increasingly being used for its living and non-living resources. With the cumulative effects of pollution from litter and waste, and climate-related impacts, the deep-sea benthic fauna are being seriously affected (Ramirez-Llodra et al., 2010). Given the impediment of fragmentary knowledge about deep-sea ecosystems, it is clear that we cannot protect what has only been sparsely sampled, mapped, and described (Glover et al., 2018; Ficetola, Canedoli & Stoch, 2019). Therefore, concerted efforts not only to incorporate but also to produce new scientific knowledge into decision-making processes are essential and not to be delayed in order to develop sustainable management practices to conserve a representative range of deep-sea habitat types and communities. In this study, we investigated macro- and megabenthic communities along the ARS in the deep Norwegian Sea. In the following, we discuss the various components of the ridge’s geological and biological diversity, starting with a characterization of the physical environment through its biotic mega- and macrofaunal components towards assessing its ecological and biological value. This will be valuable data for the Faroese, Icelandic, and Norwegian managements of the deep sea, and will support the ongoing work with describing vulnerable areas and possibly VMEs.

### Environmental and geological and settings of the Ægir Ridge System

The geologically and environmentally unique ARS offers a large variability of environment over short and long distances associated with rapid changes in depth and sea floor substrates. In its relatively shallow southern end (Grjíđargljúfur Gorge), the ARS has steep (up to 60°) and indented valley walls that do not follow typical fault lines. Similar morphologies are observed along at the head of submarine canyons, where water and turbidity current incise the continental shelf (Maier Johnson & Hart, 2018). In addition to the variations in the morphology and depth, the ARS displays variations in the substrate. Backscatter analysis has shown a change in the proportion of hard substrate associated with water depth and slope. The correlation between slope and backscatter intensity indicates that the abundance of soft substrate is related to the steepness of slopes, with no coherent soft sediment layer found on slope steeper than 30°. As the average seafloor deepens in the Norwegian Basin, the median valley become less deep compared to the surrounding seafloor and the valley walls (Ránarhryggur Ridge, Drafnarhryggur Ridge) which is typical of heavily sedimented fault scarps. There is also an increase of soft substrate in the deepest section of the ARS that can be associated with: 1/ an increase in sediment thickness: ∼200 cm at the southern rim (Straume et al., 2019), up to 1,400 cm in the valley center (Uenzelmann-Neben et al., 1992) and 2/ a decrease of the amount of pebbles (4–64 mm in diameters) and cobbles (64 to 256 mm in diameters) that cover the shallow rim of the Gríđargljúfur Gorge, which are absent in the central section N of the ridge. These fragments contribute to increase the sediment grain size and offer hard substrate for organisms. The difference of sediment thickness could indicate that the sediment is transported from the shoulders to the valley center, but also along the ARS valley from the south to north where sediments accumulate.

In addition to morphology and substrate variation, the ARS is affected by different currents and water masses. The oceanographic CTD profiles (Fig. 4) taken in the middle (North square in Fig. 1) and southwestern areas of the ARS showed the influence of different water masses of both Atlantic and Arctic origin, with the warmer, less dense Atlantic water in the upper water column and the colder, denser Arctic water in waters from the upper bathyal (600 m to 1,000 m) to abyssal range (>3,000 m). These observations were in line with the current knowledge of the oceanographic settings in the Norwegian Basin and wider Nordic seas as being a mixing zone of different water masses (*e.g.*, Semper et al., 2019; Semper et al., 2020). Especially the southern end of the ARS is influenced by several circulation systems including the Iceland-Faroe Slope Jet (IFSJ), the warm and saline North Atlantic Current (NAC), and the Faroe Current (Huang et al., 2020; Semper et al., 2020) and is still of interest for studies on sources and upstream pathways of dense overflow water in the Nordic seas and the on the AMOC (Atlantic Meridional Overturning Circulation) (Huang et al., 2020; Chafik et al., 2020). The deep and strong IFSJ on the southern tip of the ARS could be a major inducer of sediment erosion at the shallow edges of the ridge. To find evidence for this suggestion however, further studies must be made to observe local and fine scale currents.

In recent decades, the AMOC’s importance to the Earth’s climate has been identified (Boers, 2021). Studies observed its overall weakening (*e.g.*, Ceasar et al., 2018; Liu et al., 2020; Boers, 2021), as well as a stable partition of subpolar overturning in the eastern North Atlantic basins (Fu et al., 2020). Additionally, faster, warm Atlantic currents have been observed, driving the poleward extension of temperate marine fauna into the Arctic (*e.g.*, Oziel et al., 2020; Caspó, Grabowski & Weslawski, 2021), a phenomenon termed “Atlantification”. The analyses of our CTD casts showed evidence of Atlantic-origin, warmer waters in the upper layers (0–600 m) at all sites. Gislason & Silva (2012) showed how the Arctic and North Atlantic different water masses define the composition and abundance of the zooplankton communities. To study and understand the effects and extent of Atlantification, Caspó, Grabowski & Weslawski (2021) highlighted that both needed to be known, the distribution of pelagic and benthic animals in the North Atlantic/Arctic areas and the evolutionary history of the fauna. The southwestern end of the ARS is neighboring the northern end of the IFR, while the more northerly part of the ARS is rising from the abyssal Norwegian Basin (Hjartarson, Erlendsson & Blischke, 2017). This area is at the boundary of benthic cold-temperate boreal Atlantic and subpolar/polar Arctic faunal elements (*e.g.*, Olafsdóttir & Gudmundsson, 2019. Along the Norwegian shelf northwards range extensions for about 200 species in the last three decades has been observed by Narayanaswamy, Bett & Hughes (2010). Less is known on the diversity and species ranges of the bathyal and abyssal fauna of the Norwegian Basin and the ARS within.

### What type of megafauna communities do we see?

The ROV footage from the ARS showed different deep-water communities where sponges and soft corals play a role as habitat-forming elements. The images revealed the dominance of cold-water sponge fauna. Hestetun, Tompkins-Macdonald & Rapp (2017) that showed that cold-water carnivorous sponges of the family Cladorhizidae were represented with seventeen species belonging to *Cladorhiza*, *Lycopodina*, *Asbestopluma,* and *Chondrocladia* in the Greenland-Iceland-Norwegian (GIN) Seas. Many of them were close to the IFR, like the amphi-Atlantic *C.a* (*C*.) *grandis* that is distributed in the cold-water mass north and northeast of Iceland (north of the GIF) (Hestetun, Tompkins-Macdonald & Rapp, 2017). Furthermore, *Geodia hentscheli* (Cárdenas et al., 2010), observed here, was considered an Arctic species and was commonly found in the Denmark Strait and in the Nordic seas (Cárdenas et al., 2013). Other cold related species found were the hexactinellids *S. rosea* and *C. arcticus*, that were dominant in the northern part of ARS. A large-scale predictive mapping of possible distribution of VMEs in the Nordic seas show that the sponges *C. (C.) arcticus*, *Cladorhiza* sp., *C. (C.) grandis*, and *Lycopodina* sp. as well as *Geodia parva* Hansen 1885 and *G. hentscheli* are highly likely to be present in the sea north of the GSR as supported in Cárdenas et al. (2013) and Hestetun, Tompkins-Macdonald & Rapp (2017).

The sponge species and assemblages found in the ARS are characteristic of the Nordic seas or wider Arctic (Klitgaard & Tendal, 2004; Murillo et al., 2018), although a few species (*e.g.*, *C. (C). grandis*) are also found at lower latitudes, particularly in the western part of the North Atlantic (Hestetun, Tompkins-Macdonald & Rapp, 2017). Their distribution in the various sections of the ARS is very likely driven by the prevailing water masses, with the deeper areas dominated by a few structural species (*e.g.*, *C. arcticus*) and their associates adapted to comparatively colder Arctic water; and shallower areas with a more diverse megafauna likely benefiting from a dynamic mixing between water masses supplying oxygen and nutrients, as well as preventing high sedimentation. The findings in the ARS are supported by previous observations from neighboring ridges and straits (Meyer et al., 2016; Roberts et al., 2018; Meyer et al., 2019; Ramirez-Llodra et al., 2020).

The soft nephteidae corals, *Gersemia fruticosa* (Sars, 1860), *G. clavata* (Danielssen, 1887), *G. rubiformis* (Ehrenberg 1834), *Drifa glomerata* and *Duva florida* (Rathke, 1806) are widely distributed around Iceland. The records date back to the year 1900 at a depth range of 630–184 m (Jungersen, 1917; Madsen, 1944a; Madsen, 1944b), 1993 at a depth range 495–1,350 m (unpublished BIOICE data, SH Olafsdottir & S Brix 2021 pers. obs.) and most recently in 2016 and 2017 at 460–760 m depth (Olafsdóttir & Gudmundsson, 2019). Although not observed by the ROV in this survey, the sea pens *Umbellula encrinus* (Linnaeus, 1758) and *Virgularia glacialis* Kolliker 1870 have also been reported in the area at 746–790 m depth (Olafsdóttir & Gudmundsson, 2019).

As stated earlier, Arctic influenced fauna (mostly sponges) was observed during the ROV dives. No cold-water "gorgonian corals" (Alcyonacea), reef-forming corals (like *Desmophyllum pertusum* (Linnaeus, 1758) or *Madrepora oculata* Linnaeus, 1758, Scleractinia) were observed. These are, however, known from the nearby Lónsdjúp trough and the slope off Papagrunn bank (Brix et al., 2020; Ragnarsson & Burgos, 2018; Olafsdottir et al., 2020) and around the Faroe Islands (Frederiksen, Jensen & Westerberg, 1992; Tendal, 1992). Scleractinia and the various “gorgonian corals” have their distributional boundaries at the IFR and are not in the deep cold waters north of the ridge (Buhl-Mortensen et al., 2015b) although Burgos et al. (2020) models show potential occurence around the Faroes and in the Aegir Ridge area

This region is known for its strong currents and overflow regions (Hansen & Østerhus, 2000) as also indicated by the velocities in Fig. 1. We may conclude that the water bodies have an important influence shaping the operational habitat for these corals. Thus, it is important to mention here that these “typical” elements of the North Atlantic, the corals like *Paragorgia* and *Desmophyllum* were not observed along the investigated parts of the ARS. The occurrence of these corals seems to be associated with depth and suitable habitats and currents along the thermocline, which also plays an important role in other invertebrates (Høisæter, 2010; Brix et al., 2018a; Brix et al., 2018b).

For the megafauna, we have derived habitat zonation along the ARS from abyssal to the upper bathyal depth on the ridge top, recorded the presence of VME indicator taxa, especially at the steep hard-sedimented slopes. These vertical steeps of canyon-like walls had only become accessible for investigations with the availability of deep-water ROVs (Huvenne et al., 2011; Johnson et al., 2013). The soft sedimented areas of the neighboring abyssal plain or the ridge top were suitable for the investigation by towed EBS for the investigation of macrofauna.

### Macrofauna

Community composition of the soft sediment linked macrofauna in our study were largely driven by depth and to a lesser extent water mass properties. A depth zonation between a shallow (upper slope) and abyssal fauna was evident in total macrofauna. Generally, the composition of the fauna of the deep Nordic seas is influenced by cold temperatures of the prevailing water masses and the presence of the GSR, which represents a distribution barrier for fauna from the North Atlantic (Schnurr et al., 2018; Brix et al., 2018a; Jöst et al., 2019; Lörz et al., 2021; Uhlir et al., 2021). Accordingly, taxa that live in the deep Nordic basins are derived from those with broad bathymetric distributions enabling species to cross the ridge from the south (*e.g.*, Svavarsson, Strömberg & Brattegard, 1993; Weisshappel, 2000; Weisshappel, 2001). That means for peracarid crustaceans, that taxa found in the deeper parts are likely to appear shallower too, but not necessarily vice versa. Nevertheless, in our study depth had been revealed as an important factor in the differentiation of communities for all taxa investigated and at all taxonomic levels, as shown by the BIO-ENV analysis.

It has been found that many macro- and megabenthic species in the region are restricted to distinct water masses (Weisshappel & Svavarsson, 1998; Brix & Svavarsson, 2010; Schnurr et al., 2018; Lörz et al., 2018; Roberts et al., 2021). Hydrographic conditions in the region, particularly in the vicinity of the GIF, are complex and reveal considerable variation in temperature, salinity and other physical properties, that strongly shape benthic communities. The fact that we found little influence of water mass on macrofaunal patterns in our study was likely due to biologically insignificant differences between these variables; in other words, the variation in environmental parameters, such as salinity, or temperature are only minor and may have not a strong effect on faunal communities. However, the presence of certain isopod species at the upper slope sites in our study, such as *Chelator insignis* (Hansen, 1916)—a predominant North Atlantic species (Brix et al., 2014)—suggested at least an Atlantic influence for these stations. Similar to the megafauna, the deep abyssal stations, by contrast, consisted exclusively of Arctic isopod species (Svavarsson, Strömberg & Brattegard, 1993). Shallower stations were located at the lower limit of the thermocline, which was between 400 and 700 m for the region (Bett, 2001; Høisæter, 2010).

Faunal densities are often related to the amount of nutrient input and generally decrease with increasing depth (Rex et al., 2006; Rex & Etter, 2010). Patterns of total macrofauna in our study appeared to be following this trend, but only when including calanoid copepods. If copepods are removed from the analysis, a different picture emerged of elevated densities of the ARS deep-sea floor stations.

We could rule out that the high number of copepods found in each EBS sample was due to a sampling error; that is, the EBS closing mechanism worked properly at all stations and only epifauna occurred in the catch. It is likely that calanoids contained in the cold, deep Arctic water become trapped in these deeper water masses and thus “hang” off the wall of the southern end of the ARS in the Grjíđargljúfur Gorge. In reality our data offered only a small glimpse in time and space, and more sampling is needed to corroborate this pattern.

### Implications for conservation

The sustainable management and conservation of deep-sea habitats and their protection from anthropogenic pressures (bottom fishing, deep-sea mining, climate change) has been high in the global scientific and political agendas (Norwegian Ministry of Fisheries and Industry, 2019). Increasing recognition of the spatial and temporal extent of such impacts on biodiversity and ecosystem function, led to the development and implementation of area-based management tools (*e.g.*, MPA networks and EBSAs designation) and other effective conservation measures (*e.g.*, closure areas) at national and international levels.

In 2010, the Conference of the Parties (COP) to the Convention on Biological Diversity (CBD) committed to conserve at least 10% of coastal and marine areas through “effectively and equitably managed, ecologically representative and well-connected systems of protected areas and other effective area-based conservation measures” (Aichi Target 11) as part of the Strategic Plan for Biodiversity 2011–2020 (CBD, 2010). Since then, great advances have been made to identify and protect a set of ecologically or biologically significant areas, following the established EBSAs scientific criteria (SC) of: (1) uniqueness or rarity; (2) special importance for life-history stages; (3) importance for threatened, endangered or declining species and/or habitats; (4) vulnerability, fragility, sensitivity, or slow recovery; (5) biological productivity; (6) biological diversity; and (7) naturalness (CBD, 2008).

The Arctic-Intermediate Water (AIW) between 400 and 1,500 m depth over ARS are an overwintering area for the key-ecosystem species *Calanus finmarchicus* (Gunnerus, 1770) (Bagøien, Melle & Kaartvedt, 2012; Melle et al., 2014a; Melle et al., 2014b), an important resource that supports higher trophic levels (SC2). In addition, sponge aggregations and coral gardens as those found are listed as threatened and/or declining species and habitats (SC3) by the OSPAR convention (OSPAR Convention, 1992; OSPAR, 2008) and as VMEs (SC4) on account of life-history traits (*e.g.*, slow growth, high longevity) of its constituent species (FAO, 2009).

The sponge aggregations observed during our study of the ARS are similar to the ones found on the Schulz Bank located on the transition between the Mohn and Knipovich ridges (Roberts et al., 2018; Meyer et al., 2019) as well as on the Mohn’s Ridge (Ramirez-Llodra et al., 2020), and, to some extent, in the eastern Fram Strait (Meyer et al., 2016). These Arctic deep-sea habitats are known to have particular ecological significance, playing key roles both in the recycling of major nutrients (Rooks et al., 2020), and serving as refuge and nursery areas for several demersal fish species such as the Arctic skate (*Amblyraja hyperborean* [Collett, 1879]), the Roughhead grenadier (*Macrourus berglax* Lacepède, 1801) and the Greenland halibut (*Reinhardtius hippoglossoides* [Walbaum, 1792]) (Meyer et al., 2019). Unlike other types of sponge habitats, these Arctic sponge grounds, commonly known as cold-water “Ostur” are not currently protected under area-based management tools such as MPAs, either at national or international levels.

Sponge grounds are considered threatened and declining and slowly recovering. They are even considered “habitat” forming, as we observed for the rossellid sponge *Caulophacus arcticus* in the deep sedimented plains with the species interactions of pantopods, the caridean shrimp *Bythocaris,* and so far on genus or species level unidentifiable calliopiid amphipods. These amphipods are representing most probably a species new to science. Thus, the associated macrofauna—although rarely always observed in ROV video data—may play an important role and at least in the sedimented plains the little “oases” do link the macrofauna of the sedimented plain with the easily visible megafauna. The connections and species interactions still need to be studied in more detail and this may also be true for some of the sampled isopod species. Overall macrofaunal patterns will need to be further assessed and compared with other Arctic and Atlantic locations, especially with regard to the EBSA criteria, in order to identify areas with high biodiversity and a high proportion of rare or threatened species. For instance, some isopod species found in our study have been demonstrated to represent species complexes (*e.g.*, *Chelator insignis* Hansen, 1916, *Eurycope producta* GO Sars, 1866, *Oecidiobranchus nanseni* Just, 1980, *Haploniscus bicuspis* (GO Sars, 1877) as figured out in (Brix et al., 2014; Jennings et al., 2018; Schnurr et al., 2018; Paulus et al., 2022), where effective population size and range extent of undescribed species within most complexes is still unknown.

While a considerable number of MPAs and EBSAs have been designated in the North Atlantic to protect deep-sea VMEs, at present the only MPAs established in the Nordic seas fall within the Icelandic and Norwegian EEZs. Notably, none of these currently encompass sponge aggregations or soft coral gardens in their conservation targets, although some are focused on cold-water coral reefs (another VME type). Thus, the potential designation of part of the ARS as an EBSA would provide a good opportunity to augment the representativity by increasing the range of ecosystems (a ridge system) and habitats (sponge aggregations and coral gardens) under protection for a biogeographical area which is currently under-represented in the context of the wider North Atlantic. This would also be well aligned with Norwegian ongoing discussions of defining an area “the deep Norwegian Sea” including the parts of the ARS within the “Banana hole” as a “Particularly Valuable and Vulnerable Area” (Særlig verdifult og sårbart område –“SVO”) (Eriksen et al., 2021).

## Conclusions

Our research was conducted on the boundary of the Arctic Ocean close to the Atlantic Overflow, an area of complex water masses and bathymetry that is also of great importance to deep-water formation and the AMOC globally. With advancing Atlantification of the Nordic seas and accompanying effects of this and additional anthropogenic stressors (*e.g.*, pollution, fishing etc.), protection of seafloor habitats and related fauna is a pressing concern. First and foremost, however, it demands an understanding of how the fauna is structured and which factors play a role in it.

The great variation in seabed topography that defines the ARS, particularly in relation to the steep, canyon-like walls of its southern part, as well as differences in depth and water mass features let us ask if this is reflected in the diversity and composition of the macro- and megafaunal biota. Here, in particular, the use of different sampling devices provided insights into different types of fauna (macro- and megafauna) and environments (hard and soft substrate). In brief, we discovered a clear faunal zonation along the ARS from the abyssal sediment plan to the ridge top, both in macro- and megafauna communities, albeit slight variations between taxonomic groups. According to our expectations, water mass and depth were the main factors responsible for (macro-) faunal patterns. The vertical steep walls of the ridge, described as canyon-like structures, only became accessible for surveying with the availability of a deep-water ROV (Huvenne et al., 2011; Johnson et al., 2013). A biological canyon effect was evident in dense aggregates of megafaunal filter feeders and elevated macrofaunal densities. Analysis of videos and still images from the ROV also led to the discovery of a number of VME habitats and taxa in the megafaunal communities of the ARS.

As our study covered only a small portion of the ARS in two disjunct sampling areas, more work and sampling are needed to more thoroughly analyse the benthic communities along the ARS. Nonetheless, our results indicate that the depth and canyon-like topography of the ARS appear to strongly influence faunal patterns in both macro and megafauna, and further promoting the presence of VME elements. With regard to the EBSA criteria for naturalness and biodiversity, our findings are moreover in line with the ongoing calls to consider parts of the ARS as candidate locations for an EBSA.

## Supplemental Information

10.7717/peerj.13394/supp-1 Supplemental Information 1 Macrofauna standardized Standardized (1,000 m^2^ trawled area) abundances of all macrofaunal taxa collected by epibenthic sledge analysed in the study.

10.7717/peerj.13394/supp-2 Supplemental Information 2 CTD station 13

10.7717/peerj.13394/supp-3 Supplemental Information 3 CTD station 23

10.7717/peerj.13394/supp-4 Supplemental Information 4 CTD station 29

10.7717/peerj.13394/supp-5 Supplemental Information 5 CTD station 34

10.7717/peerj.13394/supp-6 Supplemental Information 6 CTD station 41

10.7717/peerj.13394/supp-7 Supplemental Information 7 CTD station 43

10.7717/peerj.13394/supp-8 Supplemental Information 8 CTD station 62

## Additional Information and Declarations

### Field Study Permissions

The following information was supplied relating to field study approvals (i.e., approving body and any reference numbers): We did sample with RV Sonne in international waters (ABNJ) and within the Icelandic EEZ during SO (MerMet 17-06). All diplomatic formulars with Iceland were received prior expedition.

### Data Availability

The following information was supplied regarding data availability: The cruise report for IceAGE3 including all station information is available in PANGEA under http://doi.org/10.48433/cr_so276. The original photographs and plates are available at figshare: Kaiser, Stefanie; Taylor, James; Lörz, Anne-Nina; Le Saout, Morgane; Schumacher, Mia; Bonk, Frederik; et al. (2022): Fig. 6 Aegir Ridge N megafaunal elements. figshare. Figure. https://doi.org/10.6084/m9.figshare.19665243.v2. Kaiser, Stefanie; Brix, Saskia; Lörz, Anne-Nina; Le Saout, Morgane; Schumacher, Mia; Bonk, Frederik; et al. (2022): Fig. 7 Aegir Ridge 2000 m to 600m. figshare. Figure. https://doi.org/10.6084/m9.figshare.19665255.v2. Kaiser, Stefanie; Brix, Saskia; Lörz, Anne-Nina; Le Saout, Morgane; Schumacher, Mis; Bonk, Frederik; et al. (2022): Fig. 8 Aegir Ridge S megafaunal elements. figshare. Figure. https://doi.org/10.6084/m9.figshare.19665267.v2. Kaiser, Stefanie; Brix, Saskia; Lörz, Anne-Nina; Le Saout, Morgane; Schumacher, Mia; Bonk, Frederik; et al. (2022): Fig. 9 Aegir Ridge N_figure_final_revised. figshare. Figure. https://doi.org/10.6084/m9.figshare.19665273.v2. Kaiser, Stefanie; Brix, Saskia; Lörz, Anne-Nina; Le Saout, Morgane; Schumacher, Mia; Bonk, Frederik; et al. (2022): Fig. 10 Aegir Ridge S_ROV3&4_final_revised. figshare. Figure. https://doi.org/10.6084/m9.figshare.19665300.v2. Kaiser, Stefanie; Brix, Saskia; Lörz, Anne-Nina; Le Saout, Morgane; Schumacher, Mia; Bonk, Frederik; et al. (2022): Fig. 6 Original photos. figshare. Media. https://doi.org/10.6084/m9.figshare.19665309.v2. Kaiser, Stefanie; Brix, Saskia; Lörz, Anne-Nina; Le Saout, Morgane; Schumacher, Mia; Bonk, Frederik; et al. (2022): Fig. 7 Original photos. figshare. Media. https://doi.org/10.6084/m9.figshare.19665318.v2. Kaiser, Stefanie; Brix, Saskia; Lörz, Anne-Nina; Le Saout, Morgane; Schumacher, Mia; Bonk, Frederik; et al. (2022): Fig. 8 Original photos. figshare. Media. https://doi.org/10.6084/m9.figshare.19665321.v2. Kaiser, Stefanie; Brix, Saskia; Lörz, Anne-Nina; Le Saout, Morgane; Schumacher, Mia; Bonk, Frederik; et al. (2022): Fig. 9 Original photos. figshare. Media. https://doi.org/10.6084/m9.figshare.19665324.v2.
