# Supplementary material for: Habitat variability and faunal zonation at the Ægir Ridge, a canyon-like structure in the deep Norwegian Sea
Source: PeerJ. 2022 Jun 15;10:e13394. doi: 10.7717/peerj.13394 (PMC9206436; doi:10.7717/peerj.13394)
Supplement: Supplemental Information 1 — Standardized (1,000 m2 trawled area) abundances of all macrofaunal taxa collected by epibenthic sledge analysed in the study. [file peerj-10-13394-s001.docx]

**S Table 1** Standardized (1000 m^2^ trawled area) abundances of all macrofaunal taxa collected by epibenthic sledge analysed in the study.
